# Asymmetric antiviral effects of ebolavirus antibodies targeting glycoprotein stem and glycan cap

**DOI:** 10.1371/journal.ppat.1007204

**Published:** 2018-08-23

**Authors:** Philipp A. Ilinykh, Rodrigo I. Santos, Bronwyn M. Gunn, Natalia A. Kuzmina, Xiaoli Shen, Kai Huang, Pavlo Gilchuk, Andrew I. Flyak, Patrick Younan, Galit Alter, James E. Crowe, Alexander Bukreyev

**Affiliations:** 1 Department of Pathology, University of Texas Medical Branch, Galveston, TX, United States of America; 2 Galveston National Laboratory, Galveston, TX, United States of America; 3 Vanderbilt Vaccine Center, Vanderbilt University Medical Center, Nashville, TN, United States of America; 4 Departments of Pathology, Microbiology and Immunology, Vanderbilt University Medical Center, Nashville, TN, United States of America; 5 Ragon Institute of MGH, MIT, and Harvard, Cambridge, MA, United States of America; 6 Department of Pediatrics (Infectious Diseases), Vanderbilt University Medical Center, Nashville, TN, United States of America; 7 Department of Microbiology and Immunology, University of Texas Medical Branch, Galveston, TX, United States of America; Division of Clinical Research, UNITED STATES

## Abstract

Recent studies suggest that some monoclonal antibodies (mAbs) specific for ebolavirus glycoprotein (GP) can protect experimental animals against infections. Most mAbs isolated from ebolavirus survivors appeared to target the glycan cap or the stalk region of the viral GP, which is the envelope protein and the only antigen inducing virus-neutralizing antibody response. Some of the mAbs were demonstrated to be protective *in vivo*. Here, a panel of mAbs from four individual survivors of ebolavirus infection that target the glycan cap or stem region were selected for investigation of the mechanisms of their antiviral effect. Comparative characterization of the inhibiting effects on multiple steps of viral replication was performed, including attachment, post-attachment, entry, binding at low pH, post-cleavage neutralization of virions, viral trafficking to endosomes, cell-to-cell transmission, viral egress, and inhibition when added early at various time points post-infection. In addition, Fc-domain related properties were characterized, including activation and degranulation of NK cells, antibody-dependent cellular phagocytosis and glycan content. The two groups of mAbs (glycan cap versus stem) demonstrated very different profiles of activities suggesting usage of mAbs with different epitope specificity could coordinate inhibition of multiple steps of filovirus infection through Fab- and Fc-mediated mechanisms, and provide a reliable therapeutic approach.

## Author summary

Recent progress in isolation of mAbs from survivors of filovirus infections suggests that the human adaptive immune system is capable of producing strong antibody responses. However, the effects of mAbs with different epitope specificity on individual steps of filovirus infection are still unclear. We evaluated a panel of mAbs obtained from survivors of natural filovirus infections, specific for the glycan cap or stem region of GP, for their effects on the attachment of viral particles to the cell surface, intracellular traffic of viral particles, proteolytic processing of GP, its interaction with the NPC1 receptor, cell-to-cell virus transmission, virus egress from infected cells, activation of natural killer cells and antibody-dependent cellular phagocytosis through Fc-mediated mechanisms. We found that antiviral activity of glycan cap-specific antibodies results from inhibition of attachment, cell-to-cell transmission and inhibition of virion budding. In contrast, the antiviral mechanisms of stem-specific antibodies were found to be inhibition of virus release from endosomal network to the cytoplasm, and also activation of natural killer cells and phagocytosis mediated by monocytes and neutrophils. The data provide new insight into the development of immune protective mechanisms during natural human infection, and have important implications for the treatment of filovirus infections by passively-transferred antibodies and vaccine design.

## Introduction

Filoviruses are enveloped, filamentous-like viruses with non-segmented RNA genome of negative polarity. The *Ebolavirus* genus of the *Filoviridae* family includes five species: Ebola (EBOV), Sudan (SUDV), Bundibugyo (BDBV), Taï Forest (TAFV) and Reston (RESTV) viruses. Most of these viruses are responsible for highly lethal disease outbreaks, for example the occurrence of 11,323 human fatalities during the 2013–2016 EBOV epidemic in West Africa [1, 2]. Despite intense international collaborative efforts, there is still no licensed therapeutic available against filovirus disease.

GP is the sole ebolavirus envelope protein responsible for cell entry and, hence, serves as the primary target for antibody-based therapies and as antigen for vaccine development [3]. The primary nucleotide sequence of the GP gene encodes soluble glycoprotein (sGP), which shares its 295 N-terminal amino acid residues with GP, whereas GP mRNA synthesis requires the insertion of an extra adenosine into the nascent mRNA via stuttering of the EBOV RNA-dependent RNA polymerase over the transcriptional editing site [4]. The mature GP at the surface of nascent virions represents a 450 kDa trimer assembled from GP1/GP2 heterodimers [3]. The GP1 subunit mediates cellular attachment of viral particles and includes base domain interacting with the GP2 subunit, and a chalice-like structure formed by the receptor-binding domain (RBD), glycan cap and heavily N- and O-glycosylated mucin-like domain (MLD). The RBD is sequestered in the chalice bowl, whereas the glycan cap and MLD are exposed and covered by a thick glycan layer that likely shields much of GP from effective humoral immune recognition [5, 6]. The GP2 subunit forms a GP stalk containing the hydrophobic internal fusion loop (IFL), two heptad repeats (HR1 and HR2), the membrane-proximal external region (MPER), the transmembrane anchor and the short cytoplasmic domain. This subunit is responsible for fusion of the viral and host cell membranes during the entry.

EBOV attachment to the cell surface occurs via two types of low affinity interactions. First, using a set of N- and O-linked glycans on the MLD and the glycan cap of GP1, virus can bind to multiple C-type lectins. Second, EBOV uses phosphatidylserine molecules incorporated into viral envelope to bind TIM/TAM receptors (reviewed in reference [7]). After adherence, virions internalize to the cell by macropinocytosis, and subsequently traffic through the labyrinth of endosomal compartments, where critical pH-dependent GP priming by cathepsin proteases takes place. The consecutive processing of GP by cathepsins L and B results in the excision of most of the GP1 subunit, which includes the glycan cap and MLD, and exposure of the RBD for interaction with the intracellular filovirus receptor, the cholesterol transporter protein NPC1 [8–11].

The first human EBOV neutralizing mAb, KZ52, was generated from RNA isolated from the bone marrow of a survivor of natural infection [12]. This mAb protected guinea pigs from lethal EBOV challenge [13], but failed to protect non-human primates (NHPs) [14]. The feasibility of post-exposure prophylaxis with antibodies in monkeys was demonstrated six years ago with total IgG purified from convalescent serum of macaques [15]. Several mAb cocktails that protect NHPs from EBOV infection have been developed subsequently: MB-003 (human or human/mouse chimeric mAbs c13C6, h13F6 and c6D8), ZMAb (murine mAbs m1H3, m2G4 and m4G7) and ZMapp (human/mouse chimeric mAbs c13C6, c2G4 and c4G7) [16, 17]. The latter cocktail, which showed a beneficial effect, however, failed to demonstrate the pre-specified statistical threshold for efficacy in a clinical trial performed during the West Africa epidemic [18]. Human mAbs from survivors of natural ebolavirus infection, rather than antibodies raised in experimentally vaccinated or infected animals, are preferable for the development of therapeutics against filovirus infections. Such antibodies have a full compatibility of Fc fragments with the receptors on human immune cells, which is expected to make them more effective due to Fc-mediated protective mechanisms. While several published studies demonstrate binding of filovirus mAbs from human survivors to GP at the atomic level [5, 19–24], none of them are characterized for the ability to affect multiple steps of viral replication. Here, we present a comprehensive comparative study of Fab- and Fc-mediated biological functions of a panel of ebolavirus mAbs from human survivors [20] targeting epitopes in the GP glycan cap and stalk region. The results indicate that both types of mAbs interfere with and target different steps of viral replication, including virus entry, egress, cell-to-cell transmission, secondary infection and facilitate destruction of infected cells through antibody-dependent cellular cytotoxicity (ADCC) and antibody-dependent cellular phagocytosis (ADCP) mechanisms. However, important differences between the two groups also were observed, suggesting complementary effects of various antibodies generated during natural filovirus infections.

## Results

### MPER-specific mAbs effectively inhibit viral entry

**Fig 1:**
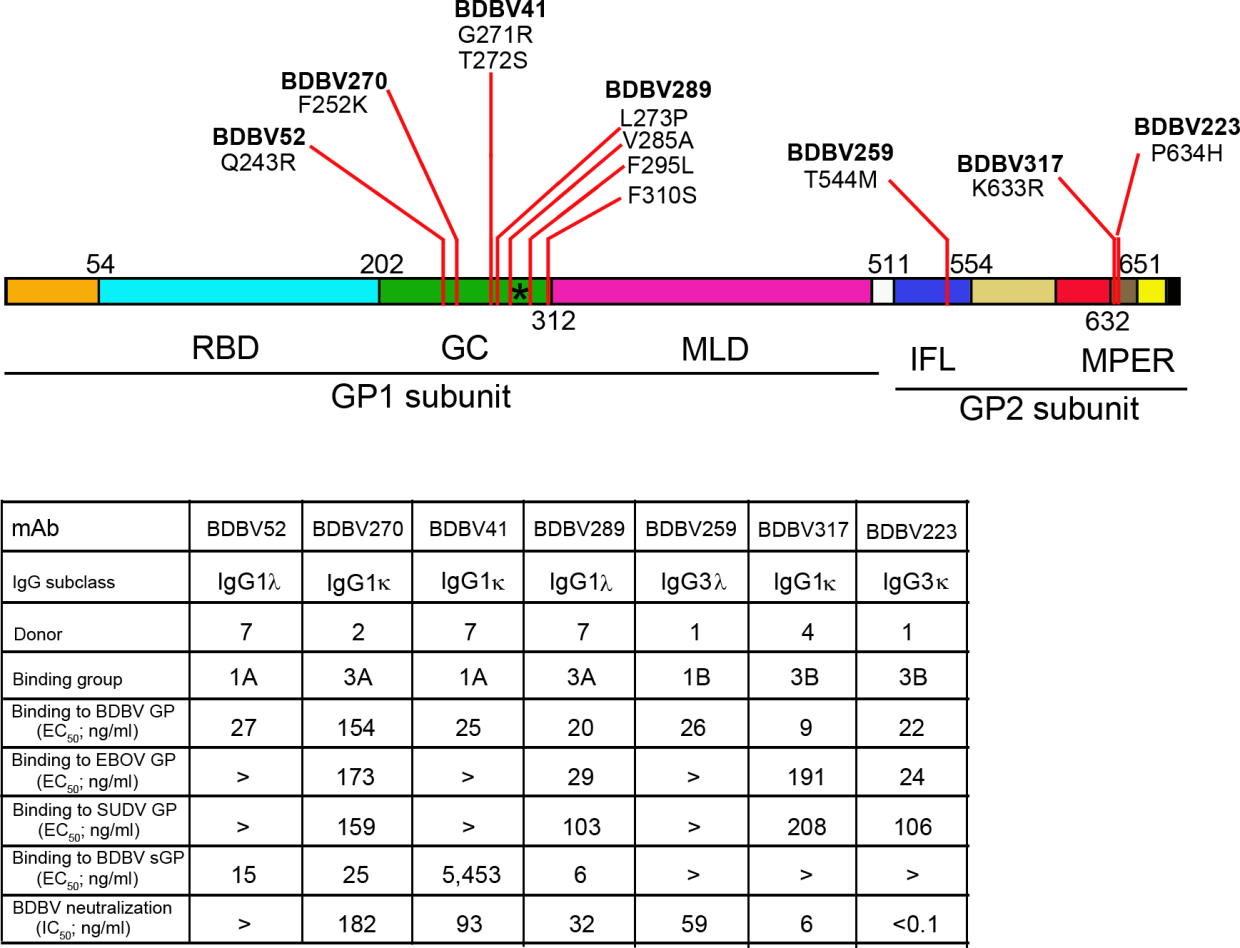
Panel of mAbs from human survivors of natural ebolavirus infections selected for the study. Schematic representation of EBOV GP domain structure: RBD, receptor-binding domain; GC, glycan cap; MLD, mucin-like domain; IFL, internal fusion loop; MPER, membrane proximal external region. Positions of the first amino acid of each domain are indicated. The black asterisk indicates the C-mannosylation site. For each antibody, positions of escape mutations [26] are indicated. EC_50_ and IC_50_ values greater than 10,000 ng/ml are indicated “>”.

In previous work, we isolated and characterized multiple mAbs from the blood of human survivors of natural BDBV infection [20]. To study mechanisms of inhibition of filovirus replication by antibodies, we selected a panel of mAbs from four donors, with differing virus neutralization properties and affinity to GPs of EBOV, BDBV and SUDV: BDBV52, BDBV270, BDBV41, BDBV289, BDBV259, BDBV317 and BDBV223 (Fig 1). Identification of epitopes demonstrated that most of the mAbs can be grouped into those recognizing two major antigenic sites: those specific for glycan cap and those specific for stem [20, 25, 26].

**Fig 2:**
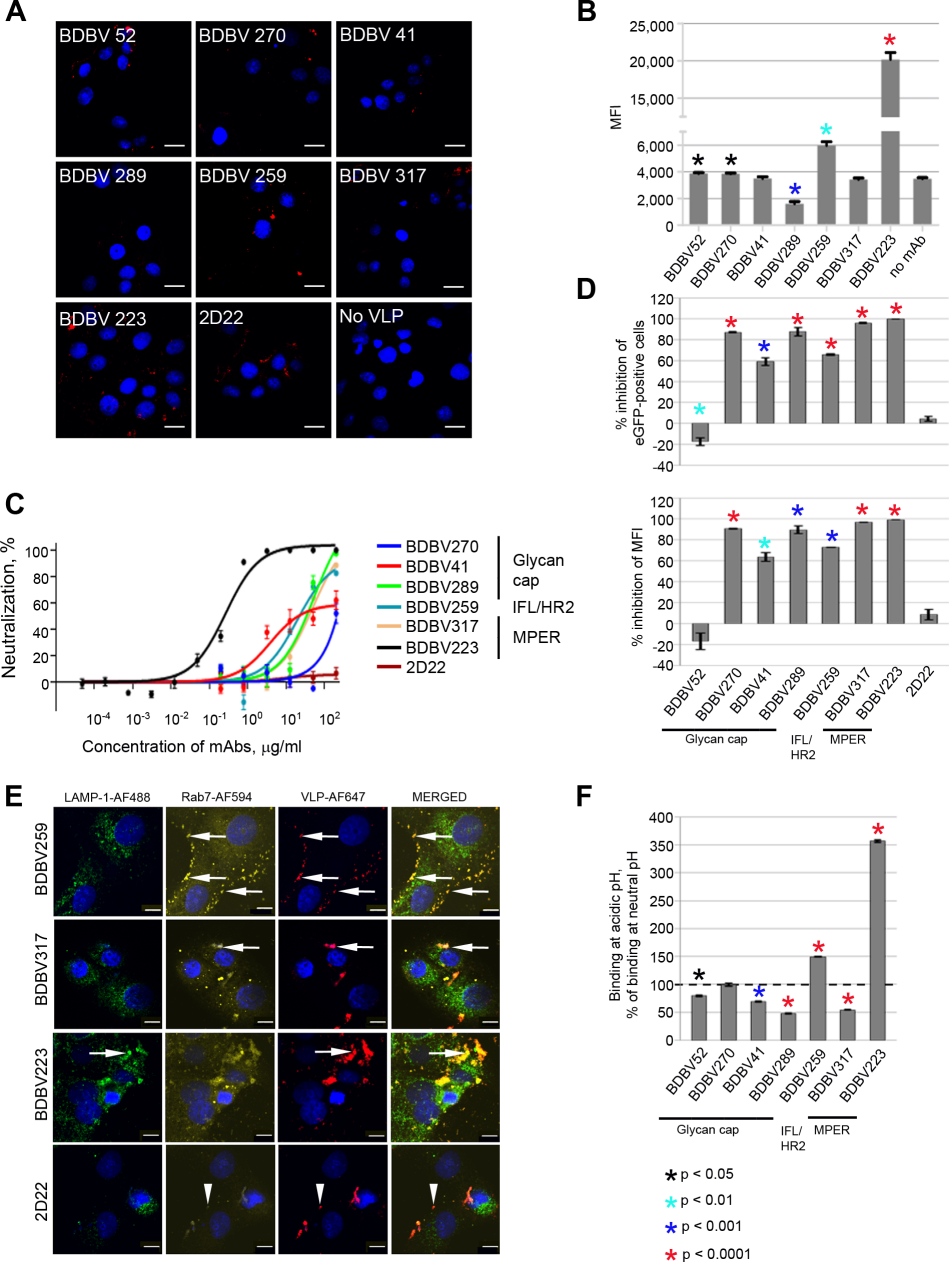
Glycan cap-specific mAbs inhibit both attachment and post-attachment steps, while stalk-specific mAbs strongly inhibit postattachment steps of viral infection. **A. Attachment of BDBV VLPs at the surface of Vero-E6 cells in presence of mAbs.** BDBV VLPs were incubated in the presence of mAbs (200 μg/ml) for 1 hour at room temperature, added to Vero-E6 cell monolayers, and cells were placed on ice for 1 hour. Then, cell-bound BDBV GP was immunostained. Red, VLPs; blue, cell nuclei. Bar = 10 μm. **B. Inhibition of EBOV/BDBV-GP_no eGFP binding to Vero-E6 cells by mAbs.** The virus was incubated with mAbs (200 μg/ml) at 37ºC for 1 hour followed by 15 min on ice and added to Vero-E6 cell monolayers. Cells were incubated for 2 hours on ice, cell-bound BDBV GP was immunostained and cells were analyzed by flow cytometry. Percentages of GP-positive cells, mean values of triplicate samples ± SE are shown. P values were calculated by unpaired Student’s t-test, compared to no mAb control. **C. Post-attachment inhibition of BDBV by mAbs.** Vero-E6 monolayers were inoculated with BDBV at 4ºC, then with four-fold serial dilutions of mAbs from 200 to 4.8 x 10^−5^ μg/ml at 4ºC, and incubated under methylcellulose overlay for 6 days at 37ºC for plaque development. Neutralization curves show percent reduction of viral plaque numbers at different mAb concentrations, mean values of triplicate samples ± SE. **D. Inhibition of virus cell entry.** Cells were inoculated with eGFP-expressing EBOV/BDBV-GP in the presence of mAbs at 100 μg/ml and analyzed by flow cytometry. Bars indicate percent reduction in the numbers of eGFP^+^ cells (top) and MFI (bottom) compared to no mAb control, mean values of triplicate samples ± SE. P values were calculated by unpaired Student’s t-test, compared to control (dengue-specific mAb 2D22). **E. Stalk mAbs trap virus inside endosomal compartments.** BDBV VLPs were incubated with 200 μg/ml of mAbs and added to Vero-E6 cell monolayers. Co-localization of VLPs (red) with the lysosomal marker LAMP1 (green) and/or late endosomal marker Rab7 (yellow) in 30 min post-inoculation, indicated by arrows. Arrowheads in the bottom row indicate the background co-localization in the presence of control mAb 2D22. Bar = 10 μm. **F. Increased binding of stalk-specific mAbs to BDBV GP in acidic conditions analyzed by ELISA.** Bars show percentages of binding at pH 5.0 compared to those at pH 7.0, mean values of triplicate samples ± SE. P values were calculated by unpaired Student’s t-test and indicate differences between antigen binding at low versus neutral pH for certain mAbs at 1 μg/ml.

To test whether any of the mAbs inhibited attachment, we incubated BDBV virus-like particles (VLPs) and mAbs at 37°C for 1 hour, added the mixtures to Vero-E6 cell culture monolayers in chambered slides, and incubated on ice for 1 hour. Then, cells were fixed, and cell-bound VLPs were immunostained. Confocal microscopic analysis of cell monolayers demonstrated a strong binding inhibition only by mAb BDBV289 (Figs 2A and S1). Agreeing with the confocal microscopy results, flow cytometric analysis demonstrated 2-fold inhibition of viral binding by BDBV289, some enhancement of binding by BDBV52, BDBV270, BDBV259, and strong enhancement for BDBV223 (Figs 2B and S2). We did not observe any enhancement of viral binding in our confocal microscopic assay for the BDBV223-treated samples. The reasons for this are unclear and may be related to the use of attached cells for the confocal microscopy and suspension cells for flow cytometry. An irrelevant human mAb 2D22 of the IgG1 isotype, specific to dengue virus envelope protein in the dimeric structure [27], was used as a control. A significant difference was not observed between the 2D22 and the no-mAb groups (S3 Fig).

For the post-attachment inhibition assay, BDBV was adsorbed first on Vero-E6 cell culture monolayers for 20 min at 4ºC. Then, mAbs were added, incubated for 20 min at 4ºC, and viral plaques were developed at 37ºC. The BDBV223 mAb strongly reduced plaque numbers, suggesting that MPER-targeting mAbs can effectively block post-attachment steps of virus replication (Fig 2C). The inhibiting effect of the other MPER-specific mAb, BDBV317, was comparable to that of BDBV289 and BDBV259 in this assay. Only marginal post-attachment inhibition was demonstrated for BDBV41 and BDBV270 mAbs from the glycan cap-targeting group. To assess the total impact of mAbs on inhibition of virus entry (binding and post-attachment steps), we used a chimeric replication-competent EBOV in which GP was replaced with its counterpart from BDBV and that expresses eGFP from an added transcriptional cassette to visualize infected cells (EBOV/BDBV-GP) [28]. EBOV/BDBV-GP was incubated with mAbs for 1 hour and adsorbed on Vero-E6 cell culture monolayers for 40 min at 4ºC. Then, cells were incubated for 24 hours at 37ºC, and the percentages of infected eGFP^+^ cells were determined by flow cytometry (Figs 2D and S4A). As expected, MPER-specific mAbs completely abolished virus entry in cells. High levels of inhibition also were demonstrated by BDBV270, BDBV41, BDBV289 and BDBV259 mAbs. Unexpectedly, the non-neutralizing BDBV52 mAb slightly increased virus entry into the cells.

### MPER- and IFL-specific mAbs trap virus inside endosomal compartments

To investigate the effect of mAbs on intracellular steps of virus life cycle, trafficking of mAb-treated VLPs through the cell organelle network was analyzed by confocal microscopy. BDBV or EBOV VLPs were mixed with mAbs, placed on Vero-E6 cell culture monolayers, incubated for 30 or 60 min and immunostained for EBOV VLPs and for endosomal markers. Unexpectedly, BDBV259 and BDBV317, but not the other mAbs, caused accumulation of VLPs in late endosomes, as evidenced by GP/Rab7 co-localization (Figs 2E, S5, S6 and S7). However, the effect of BDBV317 was relatively short, as the co-localization disappeared after one hour of incubation (S5 and S6 Figs), probably suggesting instability of BDBV317/GP complexes in the acidic pH of endosomes. When treated with BDBV223, but not the other mAbs, VLPs were found to be co-localized with the lysosomal-associated membrane protein 1 (LAMP-1) marker of lysosomes as early as 30 min after infection (Figs 2E and S6), which was still observed at 60 min (S5 and S6 Figs).

We next tested binding of mAbs to BDBV GP at low or neutral pH by ELISA (Fig 2F). Binding of BDBV317 mAb was impaired at low pH compared to neutral pH, consistent with the short duration of BDBV317/Rab7 co-localization. In contrast, binding of BDBV259 was 1.5 times higher at low pH compared to neutral pH, whereas the difference for BDBV223 mAb was as much as 3.6 times higher. Hence, we propose that an acidic pH environment stabilizes BDBV223/GP complexes, allowing this antibody to retain viral particles inside lysosomal compartments and prevent nucleocapsid entry into the cytoplasm.

We hypothesized that the accumulation in acidic compartments observed for BDBV223, BDBV317 and BDBV259 mAbs was caused by inhibition of binding of GP to NPC1 in the late endosomes. To test this hypothesis, we developed Förster resonance energy transfer (FRET) analysis using NPC1 fused to red fluorescent protein (NPC1-RFP) and GP immunostained with AlexaFluor 647. Vero-E6 cells were transfected with NPC1-RFP-expressing plasmid and incubated overnight. A modified EBOV/BDBV-GP that does not express eGFP (EBOV/BDBV-GP_no eGFP) was pre-incubated with selected mAbs for 60 min at 37ºC. NPC1-RFP-transfected Vero-E6 cell culture monolayers then were inoculated with virus-mAb complexes at an MOI of 10 PFU/cell for 2 hours, fixed, and GP was immunostained. FRET analysis was performed by scanning confocal microscopy; the NPC1-GP interaction was quantified by changes in FRET efficiency when compared with virus in the absence of mAbs (S8 Fig). We analyzed BDBV223, BDBV259 and BDBV317 in comparison with the glycan cap-specific mAb BDBV289 as a negative control, since BDBV289 inhibits attachment and entry (Fig 2A and 2B) and is expected not to reach endosomes. The FRET efficiencies for virus samples treated with mAbs were equivalent to those without mAb, suggesting that the tested antibodies did not affect binding of the virus to NPC1. We also compared the numbers of FRET-positive events, and observed a dramatic increase with BDBV223 and a more modest increase with BDBV259. The increase in the number of virus-associated events was consistent with the increased trapping of the VLPs treated with these two mAbs in endosomes (Fig 2E). Notably, BDBV223 and BDBV259, but not the other mAbs tested, were found to bind to GP at low pH (Fig 2F).

### MPER-specific, but not glycan cap-specific mAbs, neutralize thermolysin-treated virus

**Fig 3:**
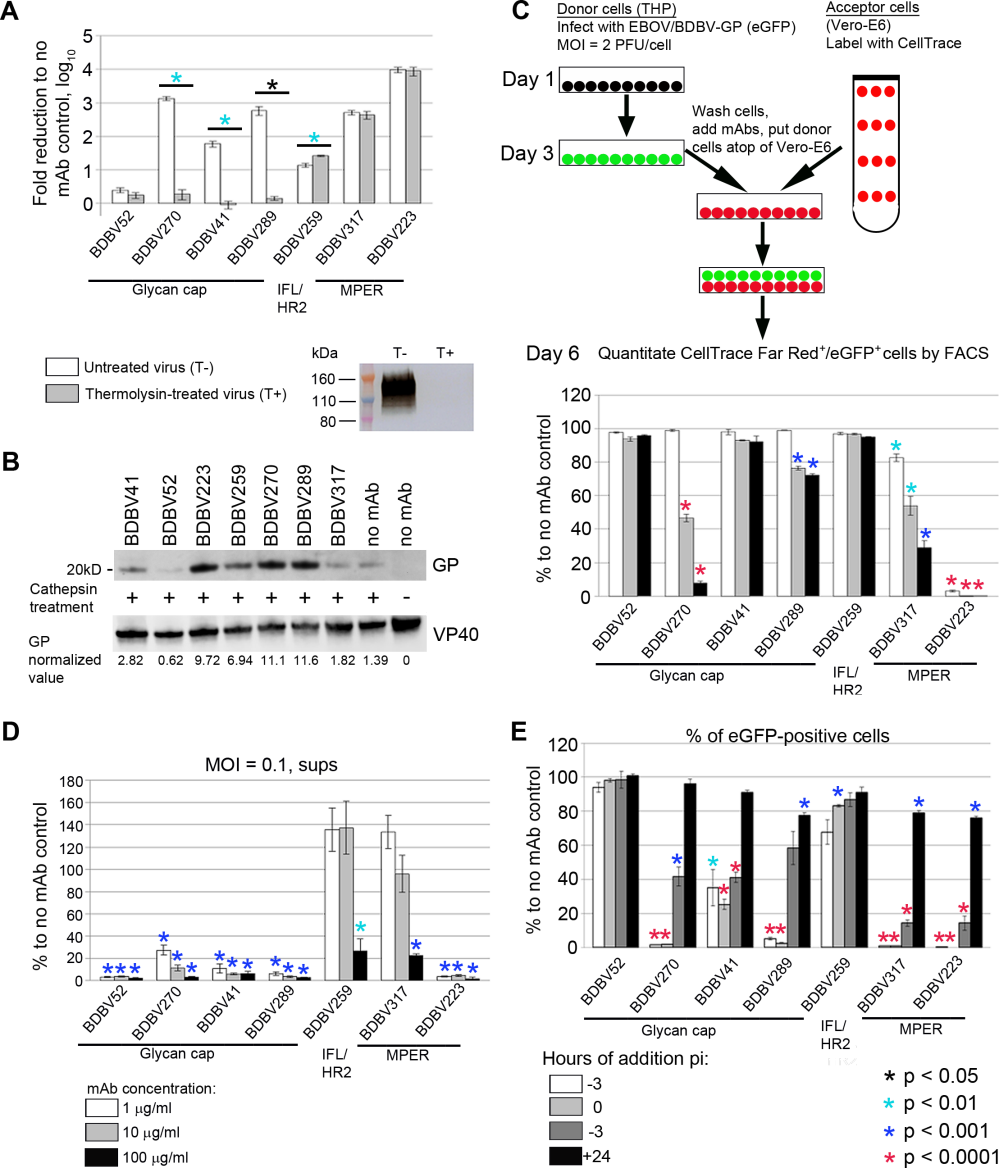
Stalk- and glycan cap-specific mAbs suppress viral infection by different mechanisms. **A. MPER-specific, but not glycan cap-specific mAbs neutralize thermolysin-treated virus.** Bars represent fold reduction of viral titers upon incubation with mAbs, mean values of triplicate samples ± SE. P values were calculated by unpaired Student’s t-test. Western blot shows elimination of the band corresponding to intact GP1 subunit after treatment of VSV/BDBV-GP particles with thermolysin. **B. MAbs do not prevent cathepsin cleavage of GP.** VLPs were incubated with indicated mAbs, treated with cathepsins B/L or mock-treated where indicated, and separated on acrylamide gel. The 20 kDa bands corresponding to cleavage product are shown. Numbers below the western blots represent GP values normalized to VP40 values. **C. Inhibition of cell-to-cell virus transmission by mAbs.** Top, schematic representation of the assay procedure. Bottom, assay results. Bars indicate percent of CellTrace Far Red^+^/eGFP^+^ cells out of total CellTrace Far Red^+^ population normalized to no mAb control, mean values of triplicate samples ± SE. P values were calculated by unpaired Student’s t-test, compared to no mAb control. The mAb concentrations are indicated in panel D. **D. Glycan cap-specific mAbs are efficient inhibitors of virus egress.** Bars indicate viral RNA load, determined by digital droplet RT-PCR, in the supernatants of cells infected with EBOV/BDBV-GP normalized to no mAb control, mean values of triplicate samples ± SE. P values were calculated by unpaired Student’s t-test, compared to no mAb control. **E. MPER-specific mAbs are more effective than glycan cap-specific mAbs when added after infection.** MAbs were added at different time points after inoculation of cells with EBOV/BDBV-GP. At 48 hours after inoculation, cells were fixed and analyzed by flow cytometry. Bars indicate percentages of eGFP^+^ cells normalized to no mAb control, mean values of triplicate samples ± SE. P values were calculated by unpaired Student’s t- test, compared to no mAb control.

As described above, GP is processed by cysteine proteases, cathepsins B and L, resulting in the removal of glycan cap and MLD from GP1 subunit followed by interaction of the exposed RBD with the C-loop of NPC1. Treatment of EBOV GP with the bacterial metalloproteinase thermolysin also results in deletion of the glycan cap and MLD, thus mimicking endosomal proteolysis of GP mediated by cathepsins [29, 30]. To test if selected mAbs interfere with late stages of virus cell entry by interacting with GP after its cleavage, we treated sucrose gradient-purified replication-competent vesicular stomatitis virus enveloped with BDBV GP (VSV/BDBV-GP) [31] with thermolysin and compared its neutralization with non-treated virus. As shown in Fig 3A, thermolysin treatment of virions resulted in complete proteolysis of GP1 subunit, and slight reduction of the virus titer (3.8-fold). Incubation of intact VSV/BDBV-GP with neutralizing glycan cap-specific antibodies led to a dramatic reduction of virus titers, whereas thermolysin-processed virus was resistant to BDBV270, BDBV41 and BDBV289, with no effect shown for the non-neutralizing BDBV52 mAb against either virus preparation. In contrast, GP cleavage with thermolysin did not reduce virus sensitivity to GP2-specific BDBV259, BDBV317 or BDBV223 mAbs, suggesting that these mAbs interact with the full-sized and processed fusion-active form of GP equally well, and, thus can inhibit multiple steps of virus entry. However, no mAb prevented the proteolysis of GP after treatment with cathepsin B and cathepsin L, as the 20 kDa GP1 fragment band resulting from the digestion of GP was present in all samples treated with cathepsin regardless of the mAb used (Fig 3B). The differences in the band intensity observed with different mAbs are probably caused by binding of mAbs to additional cathepsin cleavage sites, which are not involved in generation of the 20 kD fragment.

### MPER-specific mAbs inhibit viral transmission more effectively than glycan cap-specific mAbs

Secondary infection of cells by transfer of virions and intermediate products of viral replication (genome copies, viral proteins or the whole vRNP complexes) across the cytoplasmic bridges between infected and uninfected cells was shown to play an important role in the pathogenesis of HIV [32], influenza virus [33, 34] and EBOV [35]. Such cell-cell contacts can increase the effective viral MOI at the sites of transmission, making this route of infection spread 70-fold [35] to 2–3 orders of magnitude [32] more efficient compared to cell-free dissemination. Moreover, use of the alternative intercellular gateway for direct access to the cytoplasm of a new host cell allows virus to escape from antibodies targeting initial steps of cell entry and/or virus egress. To analyze the effects of mAbs on cell-to-cell transmission, we used a flow cytometry-based approach previously described for HIV studies [36]. THP-1 monocytes (donor cells) infected with EBOV/BDBV-GP (which expresses eGFP) were incubated with mAbs for 1 hour, placed at the top of Vero-E6 cell culture monolayers (acceptor cells) pre-stained with CellTrace Far Red, and incubated for 72 hours. Since all mAbs but BDBV52 are strong suppressors of viral entry (Fig 2D), their constant presence in the cell medium was expected to prevent spread of infection through the medium. Indeed, titration of supernatant aliquots harvested from co-cultures of THP-1 and Vero-E6 cells on days 3–5 after the inoculation of monocytes showed an absence of detectable live viral particles in samples containing BDBV270, BDBV289, BDBV223 or BDBV317 mAbs, but not in those with 2D22 or no mAb (S9 Fig). To measure cell-to-cell virus transmission, CellTrace FarRed^+^/eGFP^+^ cells were quantified by flow cytometry and expressed as percentages of the total CellTrace FarRed^+^ population (Figs 3C and S4B). Consistent with the previous experiments (Figs 2C–2F and 3A), the MPER-specific BDBV223 appeared to be the most potent mAb, as it completely suppressed the infection of acceptor cells at all concentrations tested. The other MPER-specific BDBV317, along with one glycan cap-specific BDBV270, demonstrated a clear dose-dependent inhibition of viral transmission, and the glycan cap-specific BDBV289 showed a somewhat lesser inhibition. The non-neutralizing glycan cap-specific BDBV52 mAb did not cause any detectable inhibitory effect. The overall ability of mAbs to inhibit virus transmission better corresponded to their ability to inhibit viral replication at higher (0.1 PFU/cell) than the lower (0.01 PFU/cell) MOI (S10 and S11 Figs), as was previously demonstrated for HIV [32]. The overall antibody potency in the cell-to-cell transmission assay was much lower compared to the mAb effects on primary virus entry (Fig 2D) suggesting that higher mAb doses may be required to overcome secondary virus infection through the intercellular connections.

### The non-neutralizing mAb BDBV52 inhibits viral egress

Upon completion of replication cycle inside the cell, progeny virions release into the extracellular matrix and spread the infection to bystander cells. The budding of MARV can be prevented by antibodies even in the absence of virus neutralization detectable in plaque reduction assays, possibly by bivalent cross-linking of newly formed virions to each other and to the viral proteins exposed on the cell membrane [37]. We therefore analyzed the effect of mAbs on virus release from infected Vero-E6 cells. Since the presence of the neutralizing mAbs in the medium would interfere with analysis of released viral particles by plaque assay, we quantitated both live and neutralized virus released from infected cells and present in the medium by quantifying viral genomic RNA by droplet digital RT-PCR. The egress of virus was strongly and dose-dependently inhibited by all glycan cap-specific mAbs (Fig 3D). In contrast, stalk-specific mAbs BDBV259 and BDBV317 increased the viral load in the supernatants when provided in low doses. Only high doses of these mAbs (100 μg/ml) strongly reduced release of viral particles, which did not reach, however, the level of inhibition seen for the glycan cap-specific mAbs. Taken together, these data suggest that retention of produced virions on the cells is a common mechanism of antibodies targeting external, well-exposed domains of the viral envelope proteins involved in an interaction with a cell surface. Strikingly, the single tested non-neutralizing BDBV52 mAb abolished release of virus, even at concentrations as low as 1 μg/ml. The same inhibition level was demonstrated by the highly neutralizing BDBV223 mAb, highlighting the lack of correlation between suppression of virus egress and *in vitro* neutralization.

To ensure that viral RNA detected in the cell supernatants resulted from a *bona fide* viral egress process, but not from the exit of RNA from cells *via* the exosome pathway, we conducted an additional experiment with depletion of exosomes in supernatant samples (S12 Fig). Indeed, for each of the tested mAb, regardless of its concentration, incubation of supernatants with exosome-binding beads did not result in a significant change of the viral RNA level (p > 0.05, paired Student’s t-test).

### MPER-specific mAbs are more effective than glycan cap-specific mAbs when added after infection

Next, we tested if mAbs can suppress virus replication when added at different time points after infection (Figs 3E, S4A and S13). Administration of BDBV270, BDBV289, BDBV223 or BDBV317, which belong to different epitope recognition groups, 3 hours prior to or during cell inoculation completely blocked replication of virus. Infection inhibition by BDBV41 and BDBV259 was less prominent. In contrast, the two MPER-specific mAbs BDBV317 and BDBV223 caused the strongest reduction of infected cell numbers when added up to 3 hours after inoculation. When added at 24 hours post-inoculation, none of the mAbs tested could prevent virus replication by more than 20%. Addition of BDBV52 at any time point did not change percentages of eGFP^+^ cells (Fig 3E), despite the fact that this mAb efficiently inhibited virus egress from infected cells (Fig 3D). This finding can be explained by direct virus dissemination between cells skipping the step of virion release into the extracellular space, since BDBV52 did not block cell-to-cell transmission of the virus (Fig 3C). The results indicate that MPER-specific antibodies are important for control viral replication, as they effectively prevent virus replication when added late.

### Post-translational C-mannosylation of GP protects viruses from neutralization

**Fig 4:**
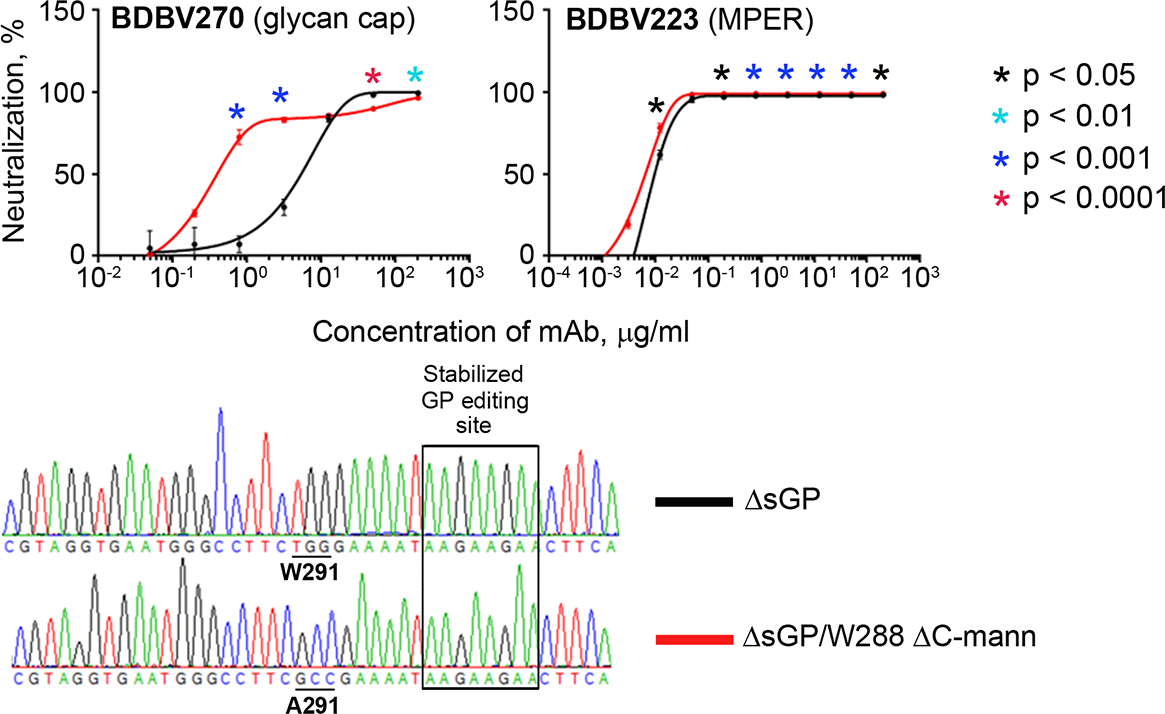
Post-translational C-mannosylation of GP reduces virus sensitivity to glycan cap mAb. Top, neutralization of EBOV/BDBV-GP ΔsGP and EBOV/BDBV-GP ΔsGP/W288 ΔC-mann constructs by representative glycan cap and MPER mAbs. The curves show percent reduction of eGFP fluorescence compared to no mAb control at various mAb concentrations, mean values of triplicate samples ± SE. P values were calculated by unpaired Student’s t-test and indicate differences between neutralization of the two viruses at certain mAb concentrations. Bottom, fragments of sequencing chromatograms of the viral genomes (positive-strand cDNA) highlighting nucleotide differences between the constructs.

Glycosylation is one of the most common posttranslational modifications of viral surface proteins. Glycosylation of viral proteins may target key epitopes at the surface of virions, masking them from antibody recognition. A decade ago, an unusual post-translational modification, C-mannosylation, was found at the residue Trp288 of EBOV sGP, which was the first demonstration of this type of glycosylation in a viral protein [38]. Since then, no evidence of any biological significance of sGP C-mannosylation has been reported. BDBV GP possesses the same W_288_AFW_291_ motif in the glycan cap, which is considered to be the most immunogenic region of filoviral GP [3, 17]. We therefore hypothesized that C-mannosylation of BDBV GP can impact antibody virus neutralization (Fig 4). To test this hypothesis, we disabled the mannosylation site in EBOV/BDBV-GP by introduction of the mutation W291A (Fig 1). To avoid interference of sGP with neutralization of viruses and to assess the pure effect of spike GP C-mannosylation on their resistance to mAbs, we also disabled the expression of sGP by stabilization of GP gene editing site [28]. BDBV270 mAb demonstrated a striking difference between neutralization of W291- and A291-bearing (ΔC-mann) mutants. At a concentration 0.8 μg/ml, the levels of neutralization of ΔsGP and ΔsGP/W288 ΔC-mann viruses by this mAb were 7.2% and 72.5%, respectively. Neutralization of the two viruses by MPER-specific BDBV223 (Fig 4) or by other mAbs included in this study performed for comparison did not show any difference. These results demonstrated for the first time shielding of a viral epitope by C-mannosylation.

### MPER-specific mAbs are more protective *in vivo* than glycan cap-specific mAbs

Next, we sought to compare protection by glycan cap- and MPER-specific antibodies. Since BDBV52, BDBV41 and BDBV259 mAbs are BDBV species-specific and do not bind EBOV GP [20], and no mouse-adapted BDBV exists so far, they were not included in animal studies. We have shown previously that BDBV289, BDBV223 and BDBV317 mAbs protect mice when given as a single 100 μg dose the day after challenge with 1,000 PFU of mouse-adapted EBOV delivered by the intraperitoneal route [20, 25]. Here, we extended the study by testing BDBV270 (S14 Fig). The *in vivo* activity of the antibody was similar to that of another glycan cap-specific mAb BDBV289 with 80% protection (4 out of 5 mice), with similar dynamics of changes in weight and disease score, although the difference in animal survival between BDBV270 and 2D22 mAb groups did not reach statistical significance (p = 0.0644, Mantel-Cox test). Thus, the two MPER-specific mAbs demonstrated complete protection and two glycan cap-specific mAbs demonstrated a high but not absolute protection in our present and previously reported studies [20, 25] with the selected dose and regiment of treatment.

### MPER-specific mAbs induce high levels of antibody-dependent cellular cytotoxicity and phagocytosis activity

Besides direct blocking of viral entry and/or exit through interaction with virions *via* Fab domains, mAbs also provide a second level of defense by cross-linking the viral proteins exposed on the surface of infected cells and Fc receptors on multiple immune cells to activate ADCC or ADCP mechanisms. Natural killer (NK) cells play a pivotal role in elimination of infected cells by ADCC. Activation of FcγRIIIa on NK cells causes the release of cytotoxic granules, which causes apoptotic death of target cells, as well as secretion of cytokines (IFNγ and TNFα) and chemokines (MIP-1α and MIP-1β), which correlate with their activation. Phagocytosis through engulfment of infected cells represents another important mechanism of rapid clearance of infection, which is mediated by FcγR-bearing immune cells including monocytes, macrophages, dendritic cells, neutrophils, and mast cells known as professional phagocytes [39].

**Fig 5:**
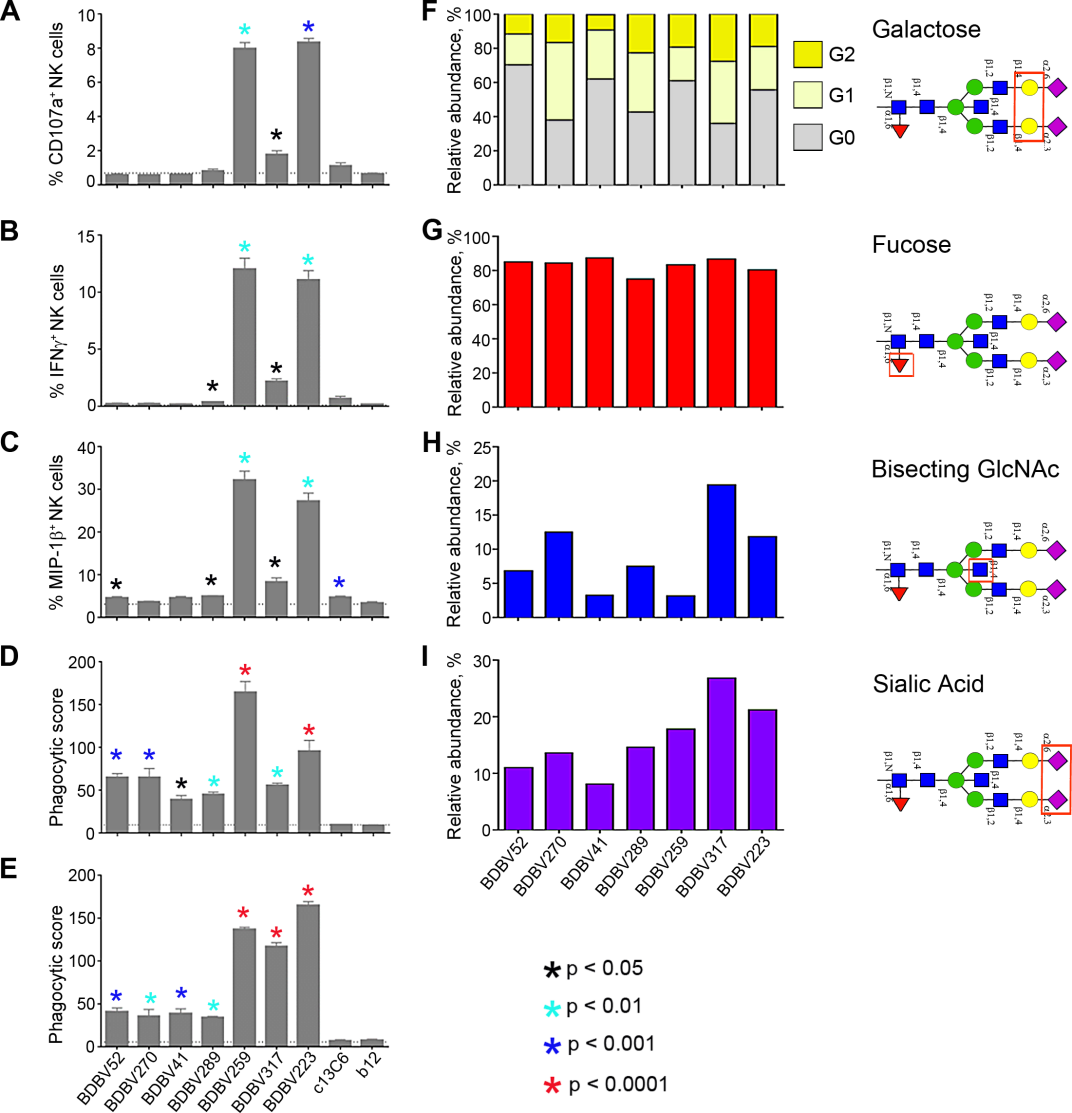
Stalk-specific, but not glycan cap-specific mAbs, induce high antibody-dependent cellular cytotoxicity and phagocytosis. **A**-**C**, expression of NK cell activation markers CD107A (**A**), IFNγ (**B**), MIP-1β (**C**), and phagocytic activity of THP-1 monocytes (**D**) or neutrophils (**E**) in response to incubation with BDBV GP immune complexes. Bars show mean values of triplicates ± SE from a single NK cell donor and are representative of two independent experiments across four NK cell donors (**A**-**C**), or mean values of duplicates ± SE, and are representative of two independent experiments (**D**, **E**). P values were calculated by factorial ANOVA (Fisher LSD test), compared to the irrelevant mAb b12, which is a HIV-specific human IgG1. The EBOV GP-specific mAb, c13C6 (IgG1), was included as a specificity control. **F**-**I**, analysis of glycan content in the Fc fragments of mAbs: the relative abundance of galactose (**F**), fucose (**G**), bisecting N-acetylglucosamine (**H**), or sialic acid (**I**). Bars show fraction of specific carbohydrate residue-containing molecules in a total antibody pool. Schematic structures of N297-associated bi-antennary glycan with analyzed sugar moieties highlighted with rectangles are indicated at the right.

Since the expression of CD107a correlates with cytokine production and cytotoxicity and it is used as a marker of NK cell degranulation [40], we used it as a marker of NK cell activation. In our experiments, the only two mAbs of IgG3 subclass, BDBV259 and BDBV223, both are stalk-specific, induced a high level of surface expression of CD107a and intracellular production of IFNγ and MIP-1β in NK cells directed against BDBV GP (Figs 5A–5C, S15A and S16A) that is consistent with the higher affinity of IgG3, compared to IgG1, for binding to FcγRs [41]. BDBV259 and BDBV223 also induced ADCP of GP-covered beads by THP-1 monocytes and neutrophils (Figs 5D, 5E, S15B, S16B and S16C). Interestingly, however, another stalk-specific mAb, BDBV317, belonging to the IgG1 subclass, showed only a slight increase in NK cell activation compared to glycan cap-specific mAbs, yet induced neutrophil phagocytosis similarly to BDBV259 and BDBV223. As interaction with FcRs can be modulated by both IgG subclass and Fc glycans structures, analysis of the glycans on the Fc domain was performed for each mAb (Fig 5F–5I). Interestingly, the stalk-specific IgG3 mAbs, BDBV259 and BDBV223, and the stalk-specific IgG1, BDBV317, were all characterized by higher sialylation of the Fc domain. As increased sialyation has been typically associated with anti-inflammatory activity [42, 43], the IgG3 subclass of BDBV259 and BDBV223 may underlie the enhanced functional activity associated with these mAbs. However, the IgG1 BDBV317 mAb was characterized by increased levels of galactose and bisecting N-acetylglucosamine (GlcNAc) glycan structures, and elevated levels of bisecting GlcNAc has been previously associated with greater phagocytic activity [44] and enhanced interaction with FcγRIIIa and ADCC activity [41]. The level of fucosylation, which negatively impacts binding of all IgG subclasses to FcγRIIIa and induction of ADCC [42], was equally high for all tested mAbs. Altogether, these data suggest that while IgG3 induced the highest level of Fc-mediated effects, the epitope location also contributed to some of the Fc-mediated effects, consistent with previously published studies with influenza virus [45–50].

### *In vivo* protection by BDBV223 MPER mAb is mediated by Fc effects

**Fig 6:**
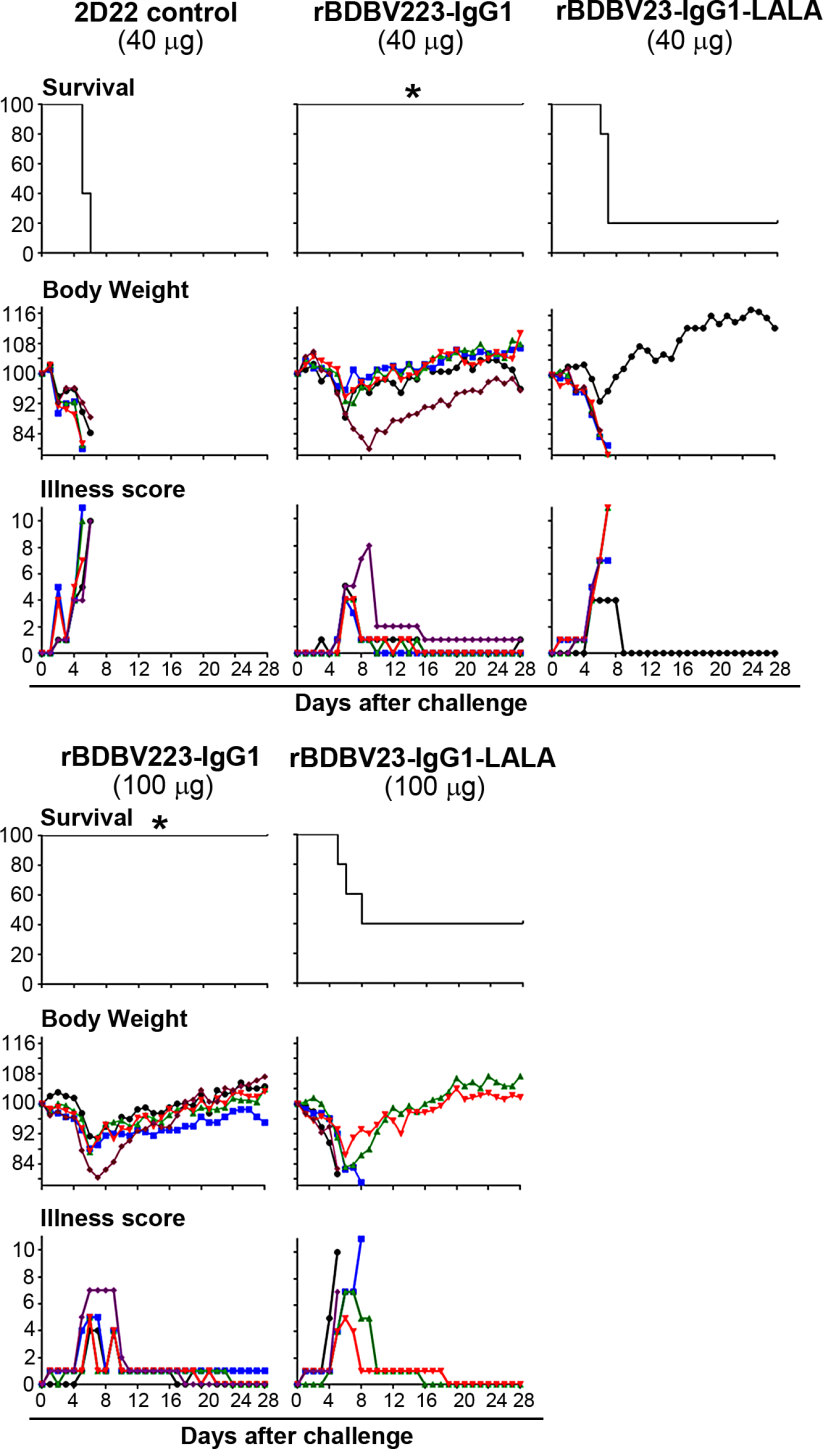
*In vivo* protection by BDBV223 MPER mAb is mediated by Fc effects. Groups of mice at five animals per group were injected with the indicated amounts of mAb by the intraperitoneal route at 24 hours after EBOV challenge. Kaplan-Meier survival curves, body weight and illness score curves are shown. *Survival difference between the rBDBV223-IgG1 and rBDBV223-IgG1-LALA recipients: 40 μg, p = 0.0158; 100 μg, p = 0.0494 (Mantel-Cox test).

Finally, we selected BDBV223 mAb, which has the broadest spectrum of inhibitory activities against different steps of viral infection *in vitro*, to address the physiological relevance of the observed Fc-mediated effects for MPER mAbs (Fig 5). We introduced the L234A/L235A (LALA) mutation, which impairs binding of antibodies to FcγRs [51–54], into the Fc region of the antibody, and compared the efficacy of mutated and non-mutated recombinant mAbs in a mouse model of EBOV infection (Fig 6). Human IgG1 and IgG3 have been shown previously to interact with mouse FcγRs [55]. Human IgG1 induces mouse innate immune effector functions at the levels equivalent to that induced by the most functional mouse subclass, IgG2a, while human IgG3 shows reduced activity with murine cells compared to human IgG1 [55]. Thus, it is possible that the human IgG3 mAbs cannot fully leverage the mouse innate immune system to maximize *in vivo* protective efficacy. We therefore generated the recombinant BDBV223 mAbs of IgG1 subclass, although the original BDBV223 subclass is IgG3 (Fig 1). Groups of BALB/c mice (5 animals per group) were inoculated with 1,000 PFU of mouse-adapted EBOV, strain Mayinga, and 24 hours later treated by the intraperitoneal route with 40 or 100 μg of wild-type rBDBV223-IgG1 or rBDBV223-IgG1-LALA. At both doses tested, wild-type antibody, but not the LALA mutant, provided complete protection of mice from the lethal EBOV infection. The differences between survival of animals in rBDBV223-IgG1 and rBDBV223-IgG1-LALA groups were statistically significant: 40 μg, p = 0.0158, 100 μg, p = 0.0494 (Mantel-Cox test). These data suggest that Fc-FcγR interactions can play a critical role in protection against EBOV infection mediated by MPER mAbs *in vivo*.

## Discussion

The unprecedented epidemic of EBOV in West Africa in 2013–2016 demonstrated the urgent need for treatments against this and related highly pathogenic filoviruses. Antibody-based therapy remains the only available effective strategy against the infection. Further progress in development of more broad and effective filovirus mAbs requires identification of the mechanism of the protective effect of these mAbs.

The glycan cap and MLD are excised by cathepsins during endosomal GP processing and, therefore they are dispensable for virus entry into the cytoplasm. It has been proposed that antibodies targeting these domains of GP are generally non-neutralizing, with some of them being able to confer protection likely through Fc-mediated mechanisms, such as ADCC or ADCP of infected cells [39]. In contrast, antibodies targeting the GP base could prevent membrane fusion [56] by blocking GP cleavage [57] or fusion-triggering conformational changes in proteolytic primed GP bound to NPC1 [58], and therefore are mostly neutralizing. However, we isolated glycan cap-specific mAbs from the blood of survivors of natural ebolavirus infection that protect mice and guinea pigs from lethal EBOV challenge [20]. Murine m8C4 mAb targeting the glycan cap was reported to neutralize EBOV and SUDV and confer partial protection of mice against these viruses; induction of ADCP by neutrophils, monocytes and dendritic cells was proposed as one of the mechanisms of protection [44]. The discovery of novel antibody epitopes in RBD [44, 59], glycan cap/RBD interface [60], IFL [19, 59, 61], and epitopes proximal to the viral membrane [19, 20] have substantially extended the concept of vulnerability sites on EBOV GP. Murine 6D6 cross-neutralizing mAb targeting the tip of the IFL prevented GP-mediated membrane fusion and protected mice against EBOV and SUDV [61]. Inhibition of cathepsin-cleaved EBOV GP binding to its endosomal receptor NPC1 was demonstrated to be the major mechanism of protection by human antibody mAb114 [57] and macaque-derived FVM04 mAb [21]. MAb114 recognizes an epitope spanning both the glycan cap and RBD, while FVM04 binds to the tip of the RBD crest. Interestingly, although antibody access to RBD is considered to be largely restricted by the surrounding glycan cap and MLD domains [5], the epitope of FVM04 is exposed in the full-sized GP. Thus, prevention of endosomal membrane fusion remains the only demonstrated mechanism of EBOV neutralization by RBD-, IFL- and GP base-specific antibodies, whereas antiviral mechanisms employed by antibodies targeting the glycan cap and novel epitopes proximal to the viral membrane are not clear.

Here, we investigated antiviral mechanisms for a diverse panel of human antibodies isolated from several human survivors of natural ebolavirus infections. Generation of escape mutant viruses resulted in mutations in the glycan cap of GP1 or in the IFL/stalk region of the GP2 subunit [26]. Glycan cap represents a well-exposed portion of the GP trimer in its native conformation, and therefore is a common target of the antibody response [5, 22], while the GP areas proximal to the viral membrane are less accessible, and have been only recently identified as a novel group of mAb epitopes [20, 62].

Since filoviruses attach to the cell surface through low-affinity interactions with multiple types of molecules, none of the filovirus-specific mAbs, including those described in the present study, were shown to completely inhibit cell attachment and infection. Moreover, all of the neutralizing mAbs studied here showed dose-dependent inhibition of viral replication when added after virus attachment to cells, suggesting they inhibit intracellular steps of entry. Interestingly, the non-neutralizing BDBV52 mAb caused an enhanced viral attachment and entry into cells, which perhaps can be mediated by the re-uptake of *de novo* synthesized viral particles retained at the cell surface by BDBV52 at the budding step.

We next analyzed mAb effects on VLP trafficking through the endosomal network. The tested mAbs did not prevent cathepsin cleavage of GP (Fig 3B) and had no effect on GP/NPC1 interaction (S8 Fig). Therefore, the co-localization of viral particles with LAMP-1 and Rab7 endosomal markers observed in the presence of stalk-binding mAbs is likely a consequence of events accompanying the merge of viral and endosomal membranes, such as conformational rearrangements of GP2 subunit after interaction of cleaved GP with NPC1.

Other than blocking of virus entry, mechanisms of restriction of infection can include inhibition of cell-to-cell transmission or budding of nascent virions from infected cells. Both steps of virus infection were found to be inhibited by glycan cap and MPER mAbs in our study. However, these mechanisms are not mutually exclusive, and, moreover, could be mediated at least in part by direct virus neutralization. The latter mechanism seems to pertain for the most potent neutralizer, BDBV223, which completely blocked virus transmission and egress, presumably by trapping it inside LAMP-1^+^ vesicles during cell entry. Unexpectedly, a comparable effect on egress inhibition was demonstrated for the non-neutralizing mAb BDBV52, with no impact on virus transmission to neighboring cells observed. Overall, mAbs demonstrated differing patterns of cell-to-cell transmission and virus egress inhibition, which could not be explained by simple differences in their neutralization activity, and is probably determined by a combination of factors, such as the location of epitope and affinity to GP at differing pH conditions.

The addition of N-linked glycans to envelope proteins is a commonly used strategy of immune evasion employed by HIV, influenza, Nipah and other viruses, which, at the same time, does not interfere with their attachment to the cell surface [63]. We found that C-mannosylation can also make virus less sensitive to a glycan cap-specific antibody. The C-mannosylation motif is located in the region shared by GP and sGP and is conserved in all known ebolavirus species: EBOV, SUDV, TAFV, BDBV and RESTV. Despite the fact that this modification was found in EBOV sGP protein [38], the results of comparative neutralization of viruses with intact or disrupted C-mannosylation site and the lack of sGP produced by the viruses used in the assay suggests that envelope GPs of BDBV, and likely of all other ebolaviruses, are also subjected to C-mannosylation. The neutralization kinetics showed that the mannose residue on W288 is likely to restrict epitope access for at least some of the glycan cap-specific mAbs.

Antibodies mediate antiviral effects both by binding epitopes on targeted pathogens by Fv region interactions and by activating Fc receptor-bearing effector cells, such as NK cells, neutrophils, macrophages and dendritic cells by Fc domain interactions. The spectrum of Fc-mediated effects induced by an antibody depends on its affinity for binding to particular FcγRs, which, in turn, depends on the IgG subclass and Fc region glycosylation. The conformational nature of the epitope recognized also impacts the efficiency of immune cell engagement. The disruption of Fc-FcγR linkage through either introduction of a D265A mutation in the Fc region or using knockout mice with disabled FcγRs leads to a complete loss of *in vivo* protection from influenza virus by broadly neutralizing HA stalk-targeting mAbs, but not by strain-specific mAbs binding to HA head domain [45, 64]. From this insight, it was interesting to observe a substantial activation of NK cells and induction of monocyte- and neutrophil-mediated phagocytosis by stalk-specific mAbs BDBV259 and BDBV223 in our study compared to the glycan cap-specific mAbs. While the observed increased activation may be due to their IgG3 subclass, which have higher affinity for FcγRIIIa and FcγRIIa compared to IgG1 antibodies [65], the stalk-specific BDBV317 IgG1 mAb also induced greater ADCP activity by neutrophils and stimulation of NK cells compared to the glycan cap-specific mAbs tested here, which also belong to the IgG1 isotype. Therefore, it is of interest to test if the direct contact between antibody-bound filovirus GP and the effector cell is required for optimal triggering of Fc mechanisms.

**Fig 7:**
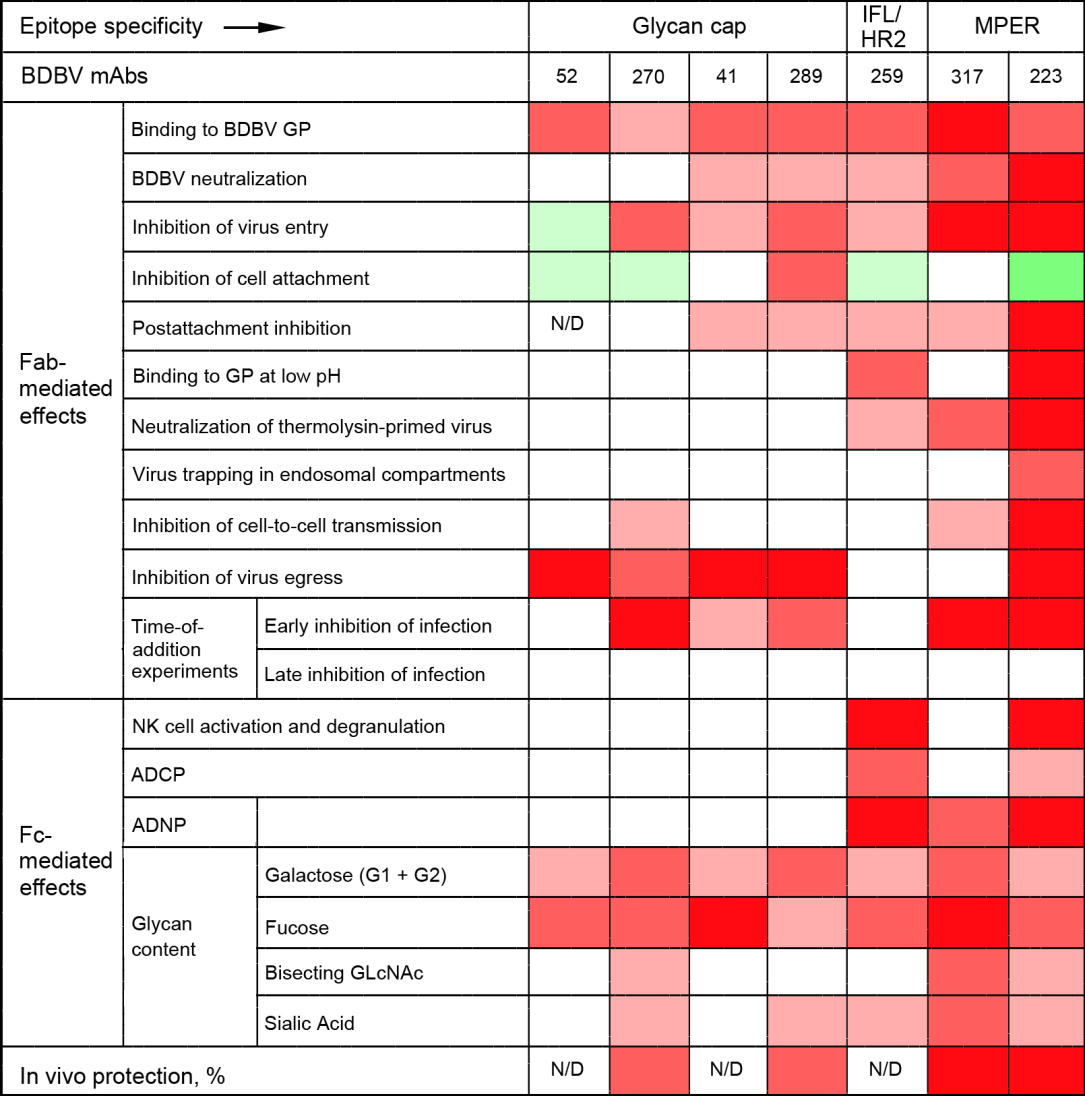
Summary of mAb effects: the greater diversity of stalk-specific mAbs compared to glycan cap-specific mAbs. Four different shades of red indicate level of inhibition, and two shades of green indicate level of enhancement, with the most intense colors corresponding to the highest inhibition or enhancement, respectively. White indicates no activity. N/D, not determined.

The biological effects of mAbs demonstrated in this study are summarized in Fig 7. In general, stalk-specific mAbs have greater Fab- and Fc-mediated effects, with the noticeable exception of the inhibition of viral egress, which was highly pronounced for all glycan cap-specific mAbs tested, and the greater level of protection *in vivo*. The current approach for treatment of filovirus infections with antibody cocktails demonstrated in animal models uses the principle of targeting of non-overlapping epitopes [20, 44, 59, 60, 66–68]; for example, our recent study demonstrated synergistic effects of the MPER-specific mAb BDBV223 and the glycan cap-specific mAb BDBV289 [20]. The data presented here suggest that there may be cooperative or synergistic effects of antibodies that block varying steps of viral replication, and cocktails based on combining such effects also should be tested. As the two contrast groups of mAbs tested in this study have different biological effects (Fig 7), the beneficial effects of cocktails of non-overlapping epitopes may be related not only to targeting different epitopes, but also to the ability of these antibodies to inhibit different steps of viral replication.

## Materials and methods

### Viruses

Wild-type BDBV, strain 200706291 Uganda, which was originally isolated from the serum of a patient during the first known outbreak [69] was passaged three times in Vero-E6 cells. The EBOV/BDBV-GP virus enveloped with glycoprotein of Bundibugyo strain, and EBOV/BDBV-GPΔsGP virus lacking sGP production were generated as described earlier [28]. To generate an EBOV/BDBV-GP derivative not expressing eGFP, the full-length clone was digested with BsiWI restriction endonuclease to remove eGFP gene, and then re-ligated. The resulting plasmid was transfected into 293T cell monolayers to rescue EBOV/BDBV-GP_no eGFP virus. To obtain EBOV/BDBV-GP ΔsGP derivative with disabled C-mannosylation site, we subjected pEBOwtΔBamHI-SbfI,AscI-PspOMI subclone with the ORF for the GP of BDBV with stabilized RNA editing site to PCR mutagenesis using the QuikChange site-directed mutagenesis kit (Stratagene). Amino acid substitution W291A in BDBV GP was introduced into the construct to disrupt C-mannosylation of W288 residue in W_288_AFW_291_ motif. For generation of full-length construct, ApaI-SacI restriction endonuclease fragment from the resulting subclone was used to replace those in pEBO-eGFP plasmid. The obtained construct was transfected into 293T cell monolayers to rescue chimeric virus with disrupted C-mannosylation site - EBOV/BDBV-GP ΔsGP/W288 ΔC-mann. Neutralization of viruses by mAbs was tested in high-throughput screening assay based on the detection of residual eGFP fluorescence [28].

### VLP generation

To generate VLPs enveloped with BDBV GP, glycoprotein ORF in pWRG7077:64755-2010-233-1_GP_optGP was substituted with that of BDBV. First, BamHI restriction endonuclease sites were disabled in pEBOwtΔBamHI-SbfI,AscI-PspOMI subclone with the ORF for the GP of BDBV with stabilized RNA editing site (ΔsGP) by introduction of silent mutations using the QuikChange site-directed mutagenesis kit (Stratagene, La Jolla, CA). Then, BDBV GP ORF was amplified from the resulting construct with following primers: direct, AGTCACGTGCGGCCGCCACC*ATGGTTACATCAGGAATTCT*; and reverse, AGTCACGTGGATCCTTATCATCA*GAGTAGAAATTTGCAAA* (the NotI or BamHI restriction endonuclease sites are underlined, and the start of the BDBV GP ORF direct sequence and the end of the BDBV GP ORF complementary sequence are italicized). The obtained PCR product was used to replace EBOV GP ORF in EBOV VLP GP plasmid by NotI and BamHI sites to get the final GP-bearing plasmid for BDBV VLP production. EBOV NP and codon optimized VP40 were cloned into the pCEZ vector [70]. pCEZ-NP was a kind gift from Drs. Kawaoka and Feldmann. The plasmids were transfected to 293T cells using TransIT-LT1 transfection reagent (Mirus). VLPs were harvested after 72 hours of the transfection, purified by sucrose gradient and quantified using ViroCyt Virus Counter (VC) 2100 (ViroCyt).

### Inhibition of virus binding to the cell surface

For confocal microscopy, Vero-E6 cell cultures (American Type Culture Collection) were grown in monolayers in chambered slides. BDBV VLPs were incubated in the presence of mAbs (200 μg/ml) for 1 hour at room temperature, added to Vero-E6 cell culture monolayers at a ratio of 500 VLP/cell, and cells were placed on ice for 1 hour. Then, cells were fixed in formalin (ThermoFisher Scientific) for 15 min, permeabilized with 0.5% Triton X-100 in phosphate buffered saline (PBS) for 15 min to increase sensitivity of the subsequent immunostaining of viral proteins. Cells then were blocked with 5% donkey serum diluted in PBS with 1% BSA and 0.1% Triton X-100 (PBS-T-BSA) for 1 hour. Next, VLPs were stained using rabbit immune serum raised against EBOV VLPs (IBT Bioservices) supplemented with rabbit polyclonal antibodies specific for BDBV GP (IBT Bioservices; all antibodies for virus staining were diluted at 1:100 in PBS-T-BSA). The slides then were incubated with donkey anti-rabbit antibodies conjugated with AlexaFluor 647 (ThermoFisher Scientific) for 1 hour at room temperature. Next, the slides were washed 3 times in PBS with 0.1% Triton X-100 (PBS-T), fixed in 10% formalin and incubated with 4',6-diamidino-2-phenylindole dihydrochloride (DAPI) (Invitrogen) at 1 μg/ml for 2 min. Then, slides were washed 5 times in PBS and mounted onto coverslips using PermaFluor mounting medium (ThermoFisher Scientific). The slides were analyzed by laser scanning confocal microscopy using an Olympus FV1000 confocal microscope housed in the Galveston National Laboratory. Lasers with 405 nm wavelength were used for DAPI excitation, and 635 nm for Alexa Fluor 647. All images were acquired using a 60x oil objective. For quantification, five representative randomly selected images were acquired and the AlexaFluor 647 fluorescence was analyzed using the FV1000 software image measurement tool. Statistical analysis was performed using ANOVA with Tukey post hoc test.

For flow cytometric analysis of virus binding, Vero-E6 cells were plated in U-bottom 96-well plates (ThermoFisher Scientific) at 10^6^ cells per well and placed on ice. EBOV/BDBV-GP_no eGFP was incubated with mAbs (200 μg/ml) at 37ºC for 1 hour followed by 15 min on ice and used to inoculate cells at an MOI of 5 PFU/cell. Cells were incubated for 2 hours on ice and washed with 2% fetal bovine serum (FBS) in PBS. Thereafter, cells were immunostained with rabbit immune serum against EBOV VLPs (IBT Bioservices) supplemented with anti-BDBV GP rabbit polyclonal antibody (IBT Bioservices); both the immune sera and antibody were added at 1:100 dilution in PBS with 2% FBS and incubated for 30 min at room temperature. After staining, cells were washed three times with 2% FBS in PBS, fixed in 10% formalin for 15 min, stained with donkey anti-rabbit antibodies labeled with Alexa Fluor 647 (ThermoFisher Scientific) and washed again 3 times with 2% FBS in PBS. Flow cytometry was performed using an LSRII Fortessa cytometer (BD Biosciences). For each sample, 10,000 events were acquired.

### Post-attachment inhibition assay

BDBV was adsorbed on Vero-E6 monolayer cell cultures in 24-well plates at an MOI of 0.1 PFU/cell for 20 min at 4ºC. Cells were washed 3 times with cold PBS, incubated with four-fold serial dilutions of mAbs for 20 min at 4ºC, washed again and covered with a 0.45% methylcellulose overlay in minimal essential medium (MEM) with 2% fetal bovine serum. Cells were incubated for 6 days at 37ºC, and plaques were visualized by immunostaining with BDBV52 mAb [20] followed by secondary goat anti-human IgG conjugated with horseradish peroxidase and 4CN two-component peroxidase substrate system (KPL). Post-attachment inhibition was calculated as a percent reduction of numbers of viral plaques developed after incubation with antibody compared to no mAb control, as previously described [71, 72]. For the no-mAb control samples, the average number of plaques per well was 263.

### Inhibition of virus entry

Three million PFU of eGFP-expressing EBOV/BDBV-GP were incubated with various mAbs at the final concentration 100 μg/ml for 1 hour at 37ºC and then adsorbed on Vero-E6 cell culture monolayers for 40 min at 4ºC. Cells were washed 3 times with MEM containing 10% FBS and incubated in fresh medium for 24 hours. Then, cells were treated with trypsin, harvested, washed twice with PBS and fixed with 4% paraformaldehyde for 24 hours for virus inactivation. Cells were analyzed by flow cytometry using an Accuri C6 cytometer (BD Biosciences) to determine the percentages of infected eGFP^+^ cells and their mean fluorescence intensity (MFI). On average, 7,728 events were acquired per sample.

### Co-localization of VLPs with endosomal markers

BDBV VLPs were generated as described above. EBOV VLPs were purchased from IBT Bioservices. BDBV or EBOV VLPs were incubated with 200 μg/ml of mAbs for 60 min at 37ºC. Monolayers of Vero-E6 cells were inoculated with VLP/mAb complexes, incubated for 30 or 60 min and fixed with 4% paraformaldehyde for 15 min. Monolayers were washed and permeabilized with 0.5% Triton-X100 solution in PBS for 15 min. Monolayers were blocked with 5% donkey serum diluted in PBS-T-BSA for 30 min. Cell monolayers were stained with mouse mAb specific for lysosomal marker LAMP-1 (Santa Cruz) at a 1:50 dilution and goat polyclonal antibodies specific for late endosome marker Rab7 (Santa Cruz) at a 1:50 dilution. VLPs were stained with rabbit immune serum against EBOV VLPs or the same rabbit immune serum supplemented with rabbit anti-BDBV GP polyclonal antibody (IBT Bioservices) at a 1:100 dilution for each antibody. Slides were incubated for 1 hour at 37ºC, washed 3 times as above, and incubated with a mixture of three secondary antibodies, each at 1:200 dilution in PBS-T-BSA: donkey anti-mouse conjugated with Alexa Fluor 488, donkey anti-goat conjugated with Alexa Fluor 594 and donkey anti-rabbit conjugated with AlexaFluor 647 (ThermoFisher Scientific). Next, cells were washed 3 times in PBS-T, and nuclei were stained with DAPI, as described above. Slides were analyzed by laser scanning confocal microscopy using an Olympus FV1000 confocal microscope with 405 nm wavelength laser for DAPI excitation, 488 nm for Alexa Fluor 488, 543 nm for Alexa Fluor 594, and 635 nm for Alexa Fluor 647.

### Binding of human mAbs to BDBV GP at neutral or acidic pH

VSV/BDBV-GP was propagated in Vero-E6 cells; at 48 hours after inoculation, the virus suspension was harvested and clarified from cell debris by low-speed centrifugation. To purify the virus, supernatants were placed atop a 25% sucrose cushion and pelleted in an ultracentrifuge for 2 hours at 175,000 x *g*, 4ºC. Pellets were resuspended in 1x STE buffer (10 mM Tris, 1 mM EDTA, 0.1 M NaCl) and further purified by ultracentrifugation in 20–60% sucrose gradient (1.5 hours at 288,000 x *g*, 4ºC). The virus-containing band was harvested, and VSV/BDBV-GP virions were washed from sucrose by final ultracentrifugation in 1x STE buffer (1 hour, 4ºC, 175,000 x *g*). The obtained viral particles were resuspended in 1x STE buffer. Flat-bottom high-binding 96-well microplates (Greiner Bio-One) were coated overnight with purified VSV/BDBV-GP particles diluted in PBS. Bound antigen was blocked with 1% bovine serum albumin (Sigma-Aldrich) in PBST buffer (0.1% Tween-20 in PBS), and treated for 20 min with 20 mM sodium citrate, pH 5.0 (Sigma-Aldrich), or PBS for 20 min. MAbs were added at 1 μg/ml in 0.1% Tween-20 containing 20 mM sodium citrate, pH 5.0, or PBST buffer, respectively, and incubated for 1 hour at 37ºC. Plates were washed three times in PBST buffer, secondary goat anti-human IgG conjugated with horseradish peroxidase (KPL) were added at a 1:2,000 dilution in PBST buffer, and plates were incubated for 1 hour at 37ºC. Next, plates were washed three times in PBST buffer, 1-component SureBlue Reserve TMB Microwell Peroxidase Substrate (KPL) was added, and plates were incubated for 20 min at room temperature and scanned in a Synergy microplate reader (BioTek) at the emission wavelength 630 nm.

### Neutralization of thermolysin-treated VSV/BDBV-GP by mAbs

VSV/BDBV-GP purified as described above was resuspended in thermolysin digestion buffer (50 mM Tris, pH 8.0, 0.5 mM CaCl_2_) and divided into two aliquots; one aliquot was treated with 0.5 mg/ml of thermolysin (Promega) and another one with an equal volume of thermolysin digestion buffer (mock-treated virus) for 40 min at 37ºC. The reactions were stopped by addition of EDTA up to the final concentration 10 mM. Virus samples were re-pelleted through a 25% sucrose cushion as described above, and washed by ultracentrifugation in 10 mM Tris, 0.1 M NaCl for 1 hour at 175,000 x *g*, 4ºC. The resulting preparations were resuspended in 10 mM Tris, 0.1 M NaCl, incubated with 100 μg/ml mAbs for 1 hour at 37ºC, or mock-incubated, and titrated on triplicate Vero-E6 cell culture monolayers using plaque reduction assay.

### Western blot analysis of thermolysin-treated VSV/BDBV-GP particles

Aliquots of thermolysin-treated or mock-treated purified virions were heated for 10 min at 95ºC and separated in Nu-PAGE 4 to 12% Bis-Tris gel with Novex Sharp Pre-Stained Protein Standard used as a molecular weight marker. Proteins were transferred to a nitrocellulose membrane using the iBlot Gel transfer system (Life Technologies). The membrane was incubated with primary rabbit polyclonal antibodies against BDBV GP (1:500; IBT Bioservices) and secondary goat anti-rabbit IgG antibodies conjugated with horseradish peroxidase (1:500; KPL). Protein bands were visualized using the chromogenic 4CN two-component peroxidase substrate system (KPL).

### Experiments with cathepsin-cleaved VLPs

EBOV VLPs alone or in the presence of 200 μg/ml of mAbs were incubated in sodium acetate buffer, pH 5.0, with 0.1 μg/μl of cathepsin B and cathepsin L at 37ºC overnight. Thereafter, VLPs were denatured in Laemmli buffer (Novex) in reducing conditions, and GP cleavage was confirmed by immunoblotting with a pan-filovirus GP-specific monoclonal antibody (IBT Bioservices). Densitometry was performed using ImageJ gel analyzer plug-in. For normalization, we used VP40 as a housekeeping protein and the 20 kDa band of GP as the target protein.

### Cell-to-cell transmission

THP-1 monocytic cells (American Type Culture Collection) were inoculated with EBOV/BDBV-GP virus expressing eGFP at MOI of 2 PFU/cell, incubated for 48 hours, washed two times to remove unbound virus, and incubated with 100 μg/ml of mAbs or no mAb. Following a one hour-long incubation, cells were placed atop of monolayers of Vero-E6 cells pre-stained with CellTrace Far Red (ThermoFisher Scientific) according to the manufacturer’s recommendations, incubated for 72 hours and fixed with 4% paraformaldehyde. Cells were analyzed by flow cytometry to determine the percentages of cells double-positive for CellTrace Far Red and eGFP of total cells positive for CellTrace Far Red. The percentage of double-positive cells indicated the percentage of cells that became infected due to cell-to-cell transmission of virus. For each sample, 30,000 events were counted. In a separate experiment, supernatant aliquots were harvested from co-cultures of THP-1 and Vero-E6 cells on days 3–5 after the inoculation of monocytes and then titrated on Vero-E6 cell monolayers.

### Virus egress assay

Vero-E6 cell culture monolayers were inoculated with EBOV/BDBV-GP expressing eGFP at an MOI of 0.1 PFU/cell, incubated for 1 hr, washed 3 times to remove non-attached viral particles, and covered with medium containing 1, 10 or 100 μg/ml of mAbs or no mAb. Cells were incubated for 48 hours, supernatants were collected, and RNA was isolated. Viral genomes were quantitated by one-step reverse transcription droplet digital RT-PCR (Bio-Rad) according the manufacturer’s instructions. Sequences of primers are available upon request. In a separate experiment, cell supernatants were incubated with exosome removal beads (Exosome-Human CD63 Isolation/Detection Reagent, ThermoFisher Scientific) for 30 min at ambient temperature, or mock-incubated, centrifuged for 5 min at low speed for sedimentation of beads, transferred to the clean tubes and subjected to RNA isolation and droplet digital RT-PCR analysis.

### Time-of-addition experiments

Vero-E6 cell culture monolayers in 24-well plates were inoculated with EBOV/BDBV-GP expressing eGFP at an MOI of 0.1 PFU/cell, with mAbs added at final concentration 100 μg/ml 3 hours prior to, at the moment of infection, or 3 or 24 hours after virus inoculation. Forty-eight hours after inoculation, cells were washed twice with PBS, treated with trypsin, harvested, fixed with 4% paraformaldehyde, and infected (eGFP^+^) cells were quantified by flow cytometry. For each sample, 10,000 events were counted.

### *In vivo* experiments

Seven-week-old BALB/c mice (Charles River Laboratories) were placed in the ABSL-4 facility of the Galveston National Laboratory. Groups of mice at five animals per group were injected intraperitoneally with 1,000 PFU of the mouse-adapted EBOV. Twenty-four hours later, animals were injected with mAbs at indicated amounts by the intraperitoneal route. Animals treated with the 2D22 mAb specific for dengue virus served as controls. The recombinant versions of BDBV223 mAb with or without LALA mutation in the Fc fragment (rBDBV223-IgG1-LALA and rBDBV223-IgG1, respectively) were generated as described elsewhere [25, 26, 73]. The animal observation procedure was performed as previously described [20]. The extent of illness was scored using the following parameters: dyspnea (possible scores 0–5), recumbence (0–5), unresponsiveness (0–5), and bleeding/hemorrhage (0–5). Moribund mice were euthanized as per the protocol approved by the UTMB Institutional Animal Care and Use Committee. The humane endpoint for weight loss was 20%. The overall observation period lasted for 28 days.

### Förster resonance energy transfer (FRET) analysis

The NPC1-encoding plasmid was purchased from OriGene. NPC1 was amplified by PCR and cloned into the p3xFLAG-CMV9 plasmid (Sigma-Aldrich). To add the red fluorescent protein (RFP) at the N-terminus, RFP gene cDNA was PCR-amplified from pcDNA3-mRFP (Addgene) and added upstream of the NPC1 coding sequence using NotI and BamHI restriction endonuclease sites. Vero-E6 cell culture monolayers were electroporated with a P3X_NPC1-RFP using Neon transfection system (ThermoFisher Scientific) with 2 pulses of 20 msec at 1,150 V, placed in chambered slides (Nalge Nunc International) and incubated overnight at 37ºC. EBOV/BDBV-GP_no eGFP was incubated with 200 μg/ml of mAbs for 1 hour at 37ºC and used for inoculation of transfected cells at an MOI of 10 PFU/cell for 30 min. Thereafter, cells were fixed with 4% paraformaldehyde for 15 min. Cell monolayers were washed 3 times in PBS-T, and viruses were incubated with rabbit immune serum against EBOV VLPs (IBT Bioservices) supplemented with rabbit anti-BDBV GP polyclonal antibody (IBT Bioservices) at a 1:100 dilution for both antibodies for 1 hour. Next, cells were washed 3 times with PBS-T and incubated with donkey anti-rabbit antibody conjugated with Alexa Fluor 647 (ThermoFisher Scientific) diluted 1:200 in PBS-T-BSA for 30 min. Next, the slides were washed 3 times in PBS-T, fixed in 10% formalin for 72 hours and removed from the BSL-4. The slides were washed 3 times in PBS and mounted onto coverslips using PermaFluor mounting medium (ThermoFisher Scientific). FRET analysis was performed by scanning confocal microscopy using an Olympus FV1000 confocal microscope with the 543 nm laser for excitation and a far-red emission filter for detection. FRET efficiency (E) was calculated using Olympus FV1000 software. The effect of mAbs on NPC1-GP interaction was measured by changes of FRET efficiency when compared with the effect of virus inoculated in the absence of mAbs.

### Antibody-mediated activation of human NK cells

Human NK cells were enriched from peripheral blood by negative selection using RosetteSep negative selection kit (Stem Cell Technologies) followed by Ficoll separation. NK cells were rested overnight in the presence of 1 ng/ml recombinant IL-15 (PeproTech). 3 μg/ml of BDBV GP (IBT Bioservices) was coated on a Maxisorp ELISA plate (Nunc) at 4°C overnight, and plates were blocked with 5% BSA prior to addition of antibodies (5 μg/ml) in PBS for 2 hours at 37°C. The control EBOV-specific mAb c13C6 was purchased from IBT Bioservices. Unbound antibodies were removed by washing wells 3X with PBS prior to addition of NK cells. The NK cells were added at 5 x 10^4^ cells/well in the presence of brefeldin A (Sigma Aldrich), GolgiStop (BD Biosciences), and anti-CD107a PE-Cy5 antibody (BD Biosciences clone H4A3) and incubated for 5 hours at 37°C. NK cells were stained with flow cytometry antibodies for the following surface markers: CD3 AlexaFluor700 (BD Biosciences clone UCHT1), CD56 Pe-Cy7 (BD Biosciences clone B159), and CD16 APC-Cy7 (BD Biosciences clone 3G8), followed by intracellular staining for IFNγ (FITC, BD Biosciences clone B27) and MIP-1β (PE, BD Biosciences clone D21-1351) to detect the production of cytokines and chemokines. Cells were analyzed by flow cytometry on a BD LSRII flow cytometer and data was analyzed using FlowJo software.

### Antibody-dependent cellular phagocytosis

Recombinant BDBV GP was biotinylated and conjugated to streptavidin-coated Alexa488 beads (Life Technologies). BDBV-coated beads were incubated with antibodies at 5 μg/ml in culture medium for 2 hours at 37°C. Human THP-1 cells (ATCC) were added at a concentration of 2.5 x 10^4^ cells/well and incubated for 18 hours at 37°C in 96-well plates. Cells were fixed with 4% paraformaldehyde and analyzed by flow cytometry on a BD LSRII using Diva software and FlowJo analysis software. The phagocytic score was determined using the following calculation: (% of AlexaFluor488^+^ cells)*(AlexaFluor488 geometric MFI of AlexaFluor488^+^ cells)/10,000.

### Antibody-dependent cellular phagocytosis

Recombinant BDBV GP was biotinylated and conjugated to streptavidin-coated Alexa488 beads (Life Technologies). BDBV-coated beads were incubated with antibodies at 5 μg/ml in culture medium for 2 hours at 37°C. Human white blood cells were isolated from peripheral blood by lysis of red blood cells using ammonium chloride potassium lysis buffer. Cells were washed with PBS, and 5.0 x 10^4^ cells/well were added to bead-antibody immune complexes, and then incubated for 1 hour at 37°C. Cells were stained with the following antibodies to identify neutrophils: CD66b Pacific Blue (BioLegend clone G10F5), CD14 APC-Cy7 (BD Biosciences clone MφP9) and CD3 AlexaFluor700 (BD Biosciences clone UCHT1). Cells were fixed with 4% paraformaldehyde and were analyzed on a BD LSRII flow cytometer. A phagocytic score was determined as described above.

### N-linked glycan pattern profiling using capillary electrophoresis

20 μg of antibodies were digested with 120 U of IDEZ (NEB) for 1 hour at 37°C to separate the F(ab′)_2_ and Fc regions. The Fc region was purified by incubating digested antibodies with magnetic protein G beads (NEB) for an additional hour at room temperature. Beads were washed with 2X with distilled water. Beads were then incubated with PNGaseF (ThermoFisher Scientific) to remove the N-linked glycan at 50°C for 1 hour. Released glycans were purified and labeled using the GlycanAssure APTS labeling kit (ThermoFisher Scientific) according to manufacturer’s instructions. Labeled glycans were analyzed on a 3500xL Genetic Analyzer (Applied Biosystems) using a POP7 polymer. Glycan peaks and relative abundance of glycan content was analyzed using the GlycanAssure Data Analysis Software v1.0 (Applied Biosystems).

### Statistics

Factorial ANOVA and two-sided t-test were used for statistical analysis of *in vitro* data. Animal survival data were analyzed by log-rank (Mantel-Cox) test.

### Ethics statement

The animal protocol for testing of mAbs in mice was approved by the UTMB Institutional Animal Care and Use Committee (protocol №1307033) in compliance with the Animal Welfare Act and other applicable federal statutes and regulations relating to animals and experiments involving animals. Challenge studies were conducted under maximum containment in an animal biosafety level 4 (ABSL-4) facility of the Galveston National Laboratory.

## Supporting information

S1 Fig Confocal microscopy analysis of the effects of mAbs on BDBV VLPs attachment to Vero-E6 cells (related to Fig 2A). **A**, Quantitative analysis of fluorescence intensity: mean values of triplicate samples ± SE. Difference compared to 2D22 mAb: * p < 0.05 (ANOVA, Tukey post hoc test). **B**, Confocal microscopy analysis of binding: a representative panel. Red, VLPs; blue, cell nuclei. Bar = 10 μm.
(PDF)

S2 Fig (Related to Fig 2B). **Binding of EBOV/BDBV-GP_no eGFP to Vero-E6 cells in presence of BDBV223 or BDBV289 analyzed by flow cytometry.** Treatments and MFI of GP-stained cells are indicated in each graph.
(PDF)

S3 Fig (Related to Fig 2B). **Binding of EBOV/BDBV-GP_no eGFP to Vero-E6 cells in presence of a non-specific mAb 2D22: comparison to no mAb control.** Cell-bound BDBV GP was immunostained and cells were analyzed by flow cytometry. Percentages of GP-positive cells, mean values of triplicate samples ± SE. P values were calculated by unpaired Student’s t-test.
(PDF)

S4 Fig Gating strategy for the flow cytometry experiments presented in Figs 2D and 3E (**A**), and Fig 3C (**B**).
(PDF)

S5 Fig Effects of mAbs on virus intercellular distribution. Cells were inoculated with BDBV VLP/mAb mixtures, incubated for 60 min and fixed. Red, VLPs; green, lysosomal marker LAMP-1; yellow, late endosomal marker Rab7; the co-localizations are indicated by arrows. Arrowheads indicate background co-localization in the presence of the irrelevant mAb 2D22. Bar = 10 μm.
(PDF)

S6 Fig Effects of mAbs on virus cell trafficking. Cells were inoculated with EBOV VLP/mAb mixtures, incubated for 30 (top) or 60 (bottom) min and fixed. Red, VLPs; green, lysosomal marker LAMP-1; yellow, late endosomal marker Rab7; the co-localizations are indicated by arrows. Arrowheads indicate rare background co-localization events in presence of the irrelevant mAb 2D22. Bar = 10 μm.
(PDF)

S7 Fig (Related to Fig 2E). **Stalk mAbs trap virus inside endosomal compartments.** Co-localization of BDBV VLPs (red) with the lysosomal marker LAMP-1 (green) and/or late endosomal marker Rab7 (yellow) at 30 min post-inoculation, indicated by arrows. Panels from two independent experiments are shown. Bar = 10 μm.
(PDF)

S8 Fig Effects of mAbs on interaction of GP with NPC1. **A.** Schematic representation of FRET for analysis of the binding of GP to NPC1 in the late endosomes. **B.** FRET efficiency, which represents a percentage of the maximal amount of fluorescence emitted by acceptor fluorophore when excited by the donor fluorophore in the presence of the indicated mAb. Cells transfected with NPC1-RFP were inoculated with EBOV/BDBV-GP_no eGFP in the presence or absence of mAbs, fixed and stained for GP. Each symbol represents an individual FRET positive event. Horizontal lines correspond to the average values of FRET positive events ± SE. The numbers of FRET positive events are shown on the top of each group. Comparison of FRET efficiency to no mAb control showed no statistical significance (Factorial ANOVA, Fisher LSD test).
(PDF)

S9 Fig (Related to Fig 3C). Inhibition of cell-to-cell virus transmission by mAbs: titration of virus in supernatants. Supernatant aliquots were harvested from co-cultures of THP-1 and Vero-E6 cells on days 3–5 after the infection of monocytes and titrated on Vero-E6 cell monolayers. Mean values of triplicate samples ± SE are shown. The limit of detection (2 log_10_) is indicated by the dotted line.
(PDF)

S10 Fig Dose-dependent inhibition of viral infection by mAbs analyzed by flow cytometry. Vero-E6 cells with various mAb concentrations in medium were inoculated with EBOV/BDBV-GP at MOI of 0.01 PFU/cell (top) or 0.1 PFU/cell (bottom), incubated for 48 hours, fixed and analyzed by flow cytometry. Bars show percentage of reduction of the numbers of eGFP^+^ cells (left) or MFI (right) compared to no mAb control, mean values of triplicate samples ± SE. P values were calculated by unpaired Student’s t-test, compared to no mAb control.
(PDF)

S11 Fig Dose-dependent inhibition of viral infection by mAbs analyzed by UV microscopy. Vero-E6 cells with various mAb concentrations in the medium were inoculated with EBOV/BDBV-GP at MOI of 0.01 PFU/cell (left) or 0.1 PFU/cell (right), incubated for 48 hours and analyzed by UV microscopy.
(PDF)

S12 Fig (Related to Fig 3D). **Exosome depletion does not affect the content of viral RNA in cell supernatants.** Bars indicate viral RNA load, determined by digital droplet RT-PCR, in supernatants of cells infected with EBOV/BDBV-GP with or without exosome depletion. Mean values normalized to no-mAb control based on triplicate samples ± SE.
(PDF)

S13 Fig MPER-specific mAbs are more effective than glycan cap-specific mAbs when added after infection. Vero-E6 cells were inoculated with EBOV/BDBV-GP at MOI of 0.1 PFU/cell, and mAbs were added at the indicated time points with final concentration of 100 μg/ml. UV microscopy photographs of cell culture monolayers taken at 48 hours after infection.
(PDF)

S14 Fig Glycan cap-specific BDBV270 mAb protects mice from lethal EBOV infection. Groups of mice at five animals per group were injected with 100 μg of an irrelevant mAb 2D22 or BDBV270 by the intraperitoneal route at 24 hours after EBOV challenge. Kaplan-Meier survival curves (p = 0.0644, Mantel-Cox test), body weight and illness score curves are shown.
(PDF)

S15 Fig Schemes of NK cell activation (**A**, related to Fig 5A, 5B and 5C) and phagocytosis assays (**B**, related to Fig 5D and 5E).
(PDF)

S16 Fig Flow cytometry gating strategy for analysis of effector functions. Gating strategy for analysis of antibody dependent activation of NK cells (**A**, related to Fig 5A, 5B and 5C), antibody dependent monocyte phagocytosis (**B**, related to Fig 5D) and antibody dependent neutrophil phagocytosis (**C**, related to Fig 5E). Representative flow cytometry plots of the indicated monoclonal antibodies in response to recombinant BDBV GP.
(PDF)
