# Supplementary figures and images for: Asymmetric antiviral effects of ebolavirus antibodies targeting glycoprotein stem and glycan cap
Source: PLoS Pathog. 2018 Aug 23;14(8):e1007204. doi: 10.1371/journal.ppat.1007204 (PMC6107261; doi:10.1371/journal.ppat.1007204)

## S1 Figure

**A**

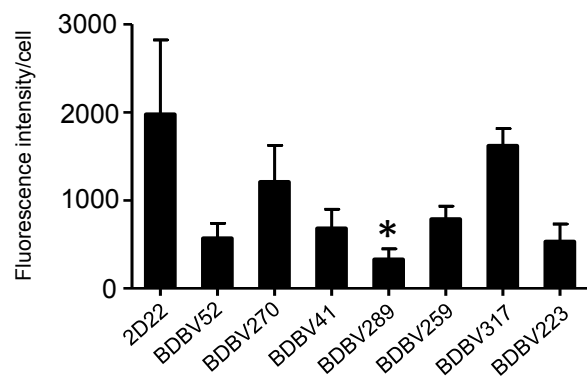

**B**

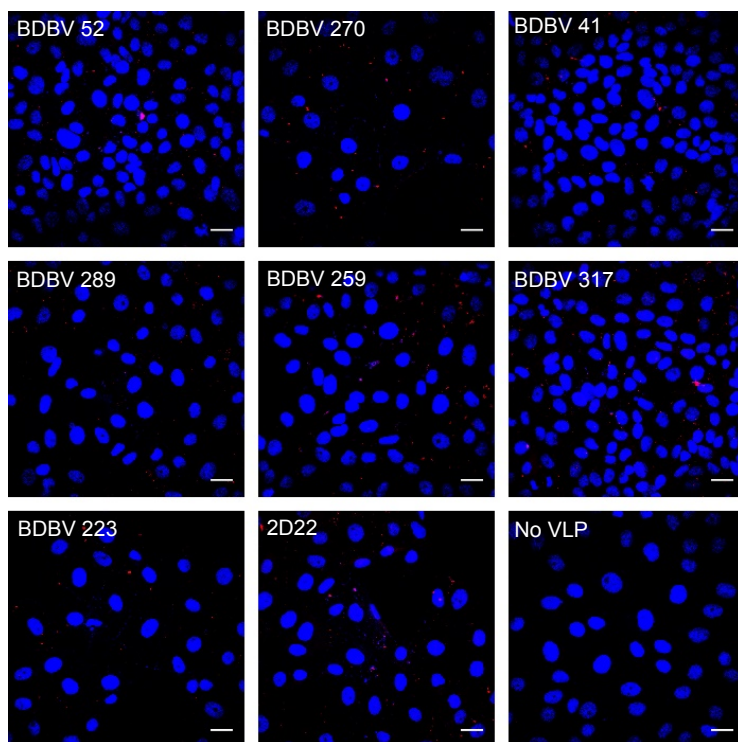

Supplement: S1 Fig — A, Quantitative analysis of fluorescence intensity: mean values of triplicate samples ± SE. Difference compared to 2D22 mAb: * p < 0.05 (ANOVA, Tukey post hoc test). B, Confocal microscopy analysis of binding: a representative panel. Red, VLPs; blue, cell nuclei. Bar = 10 μm. (PDF) [file ppat.1007204.s001.pdf]

## S2 Figure

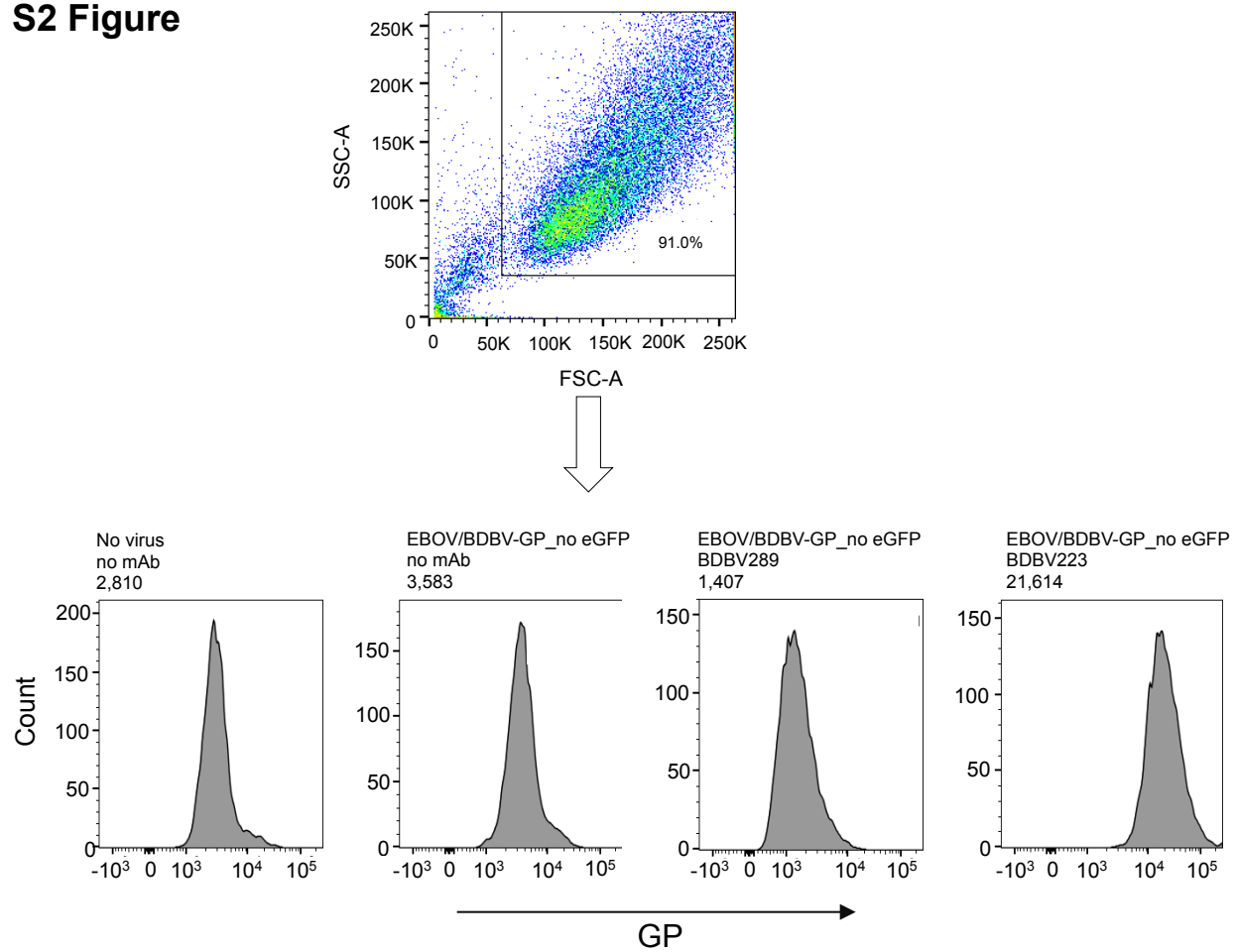

Supplement: S2 Fig — Binding of EBOV/BDBV-GP_no eGFP to Vero-E6 cells in presence of BDBV223 or BDBV289 analyzed by flow cytometry. Treatments and MFI of GP-stained cells are indicated in each graph. (PDF) [file ppat.1007204.s002.pdf]

**S3 Figure**

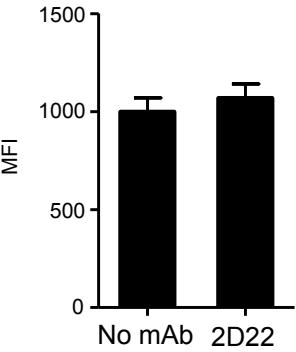

Supplement: S3 Fig — Binding of EBOV/BDBV-GP_no eGFP to Vero-E6 cells in presence of a non-specific mAb 2D22: comparison to no mAb control. Cell-bound BDBV GP was immunostained and cells were analyzed by flow cytometry. Percentages of GP-positive cells, mean values of triplicate samples ± SE. P values were calculated by unpaired Student’s t-test. (PDF) [file ppat.1007204.s003.pdf]

**S4 Figure**

**A**

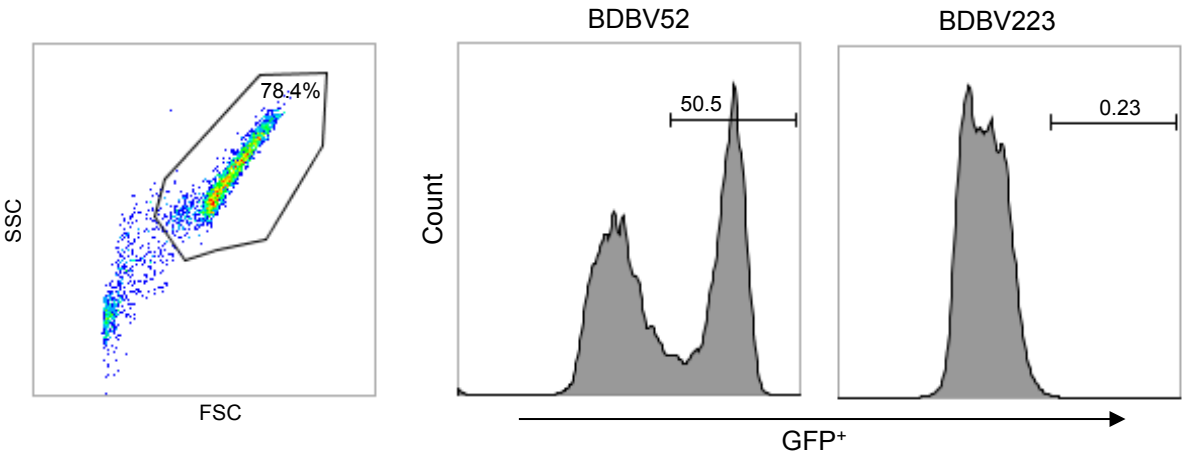

**B**

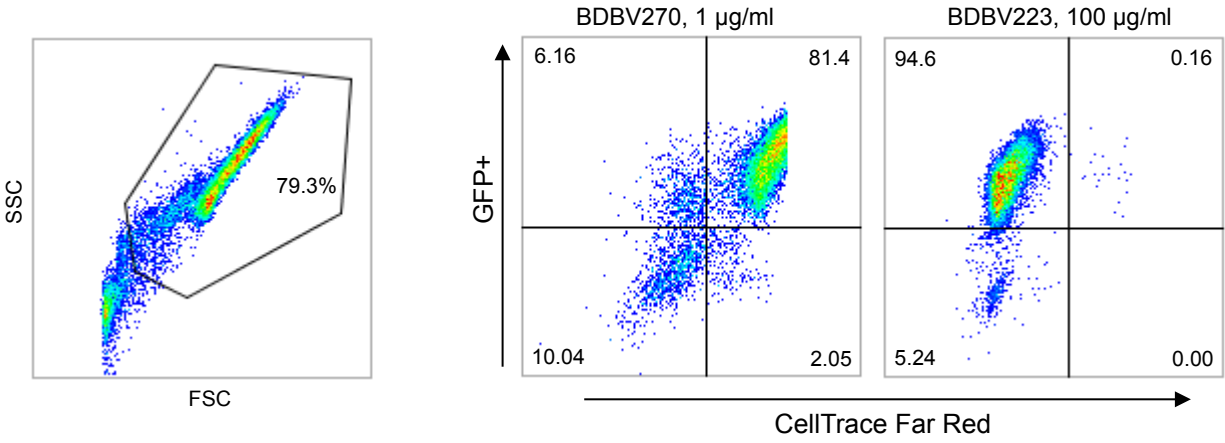

Supplement: S4 Fig — Gating strategy for the flow cytometry experiments presented in Figs 2D and 3E (A), and Fig 3C (B). (PDF) [file ppat.1007204.s004.pdf]

## S5 Figure

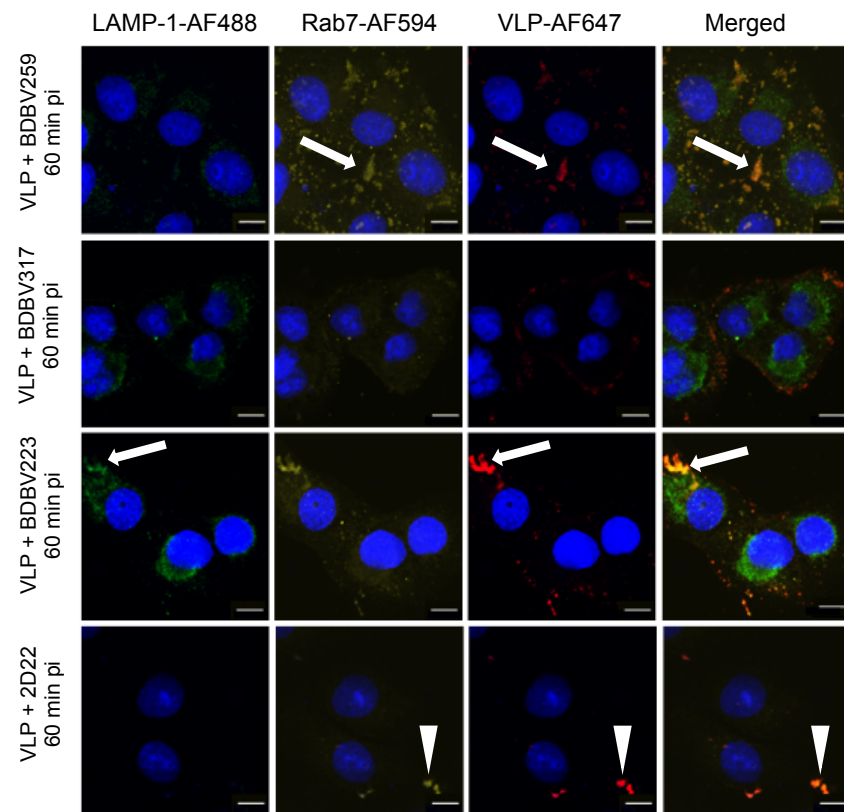

Supplement: S5 Fig — Cells were inoculated with BDBV VLP/mAb mixtures, incubated for 60 min and fixed. Red, VLPs; green, lysosomal marker LAMP-1; yellow, late endosomal marker Rab7; the co-localizations are indicated by arrows. Arrowheads indicate background co-localization in the presence of the irrelevant mAb 2D22. Bar = 10 μm. (PDF) [file ppat.1007204.s005.pdf]

S6 Figure

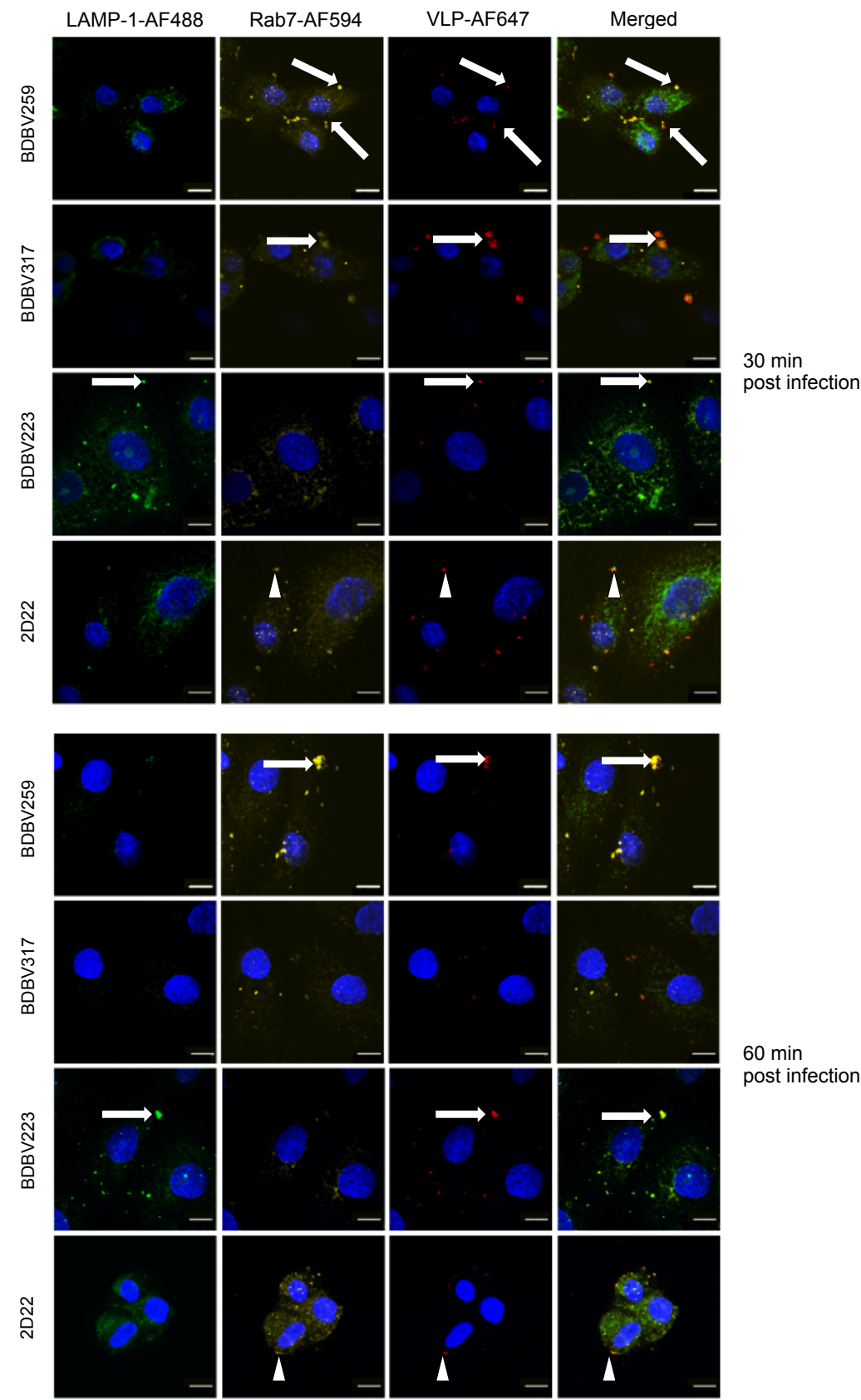

Supplement: S6 Fig — Cells were inoculated with EBOV VLP/mAb mixtures, incubated for 30 (top) or 60 (bottom) min and fixed. Red, VLPs; green, lysosomal marker LAMP-1; yellow, late endosomal marker Rab7; the co-localizations are indicated by arrows. Arrowheads indicate rare background co-localization events in presence of the irrelevant mAb 2D22. Bar = 10 μm. (PDF) [file ppat.1007204.s006.pdf]

S7 Figure

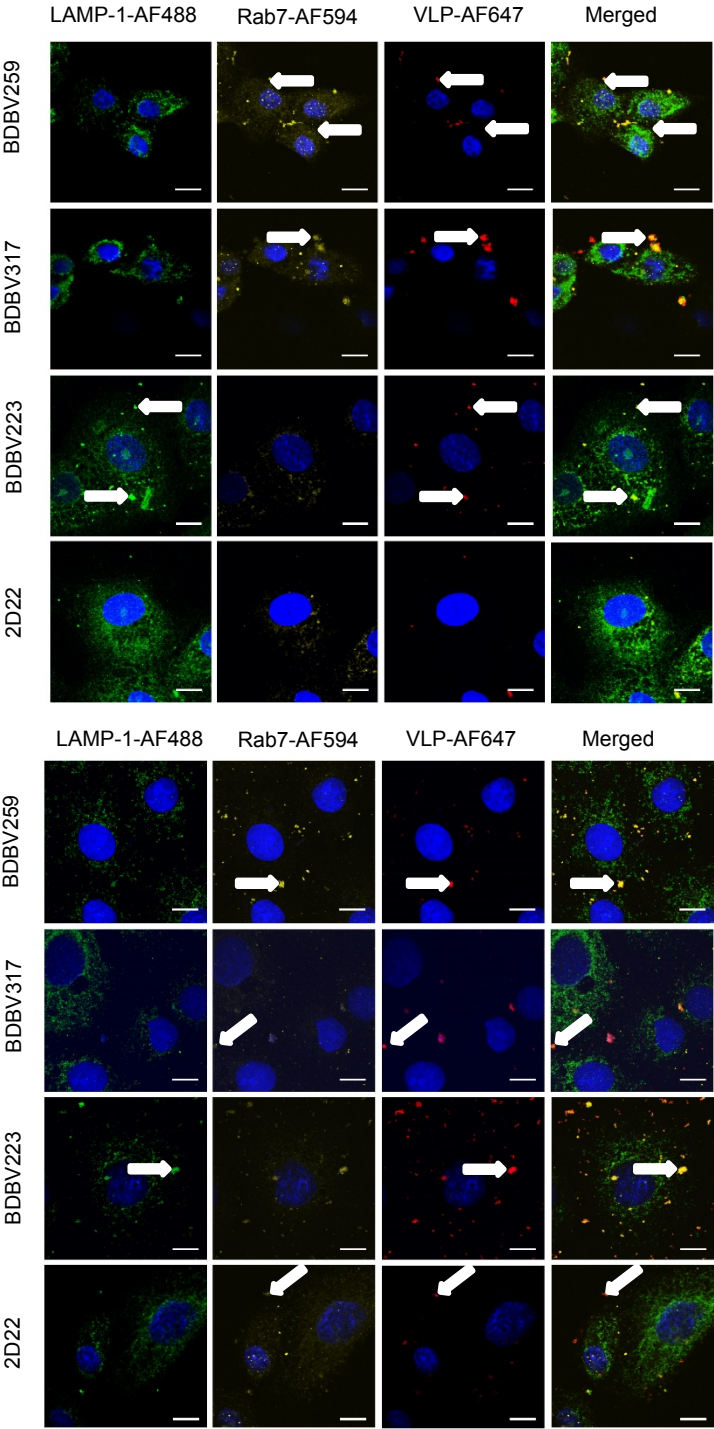

Supplement: S7 Fig — Stalk mAbs trap virus inside endosomal compartments. Co-localization of BDBV VLPs (red) with the lysosomal marker LAMP-1 (green) and/or late endosomal marker Rab7 (yellow) at 30 min post-inoculation, indicated by arrows. Panels from two independent experiments are shown. Bar = 10 μm. (PDF) [file ppat.1007204.s007.pdf]

S8 Figure

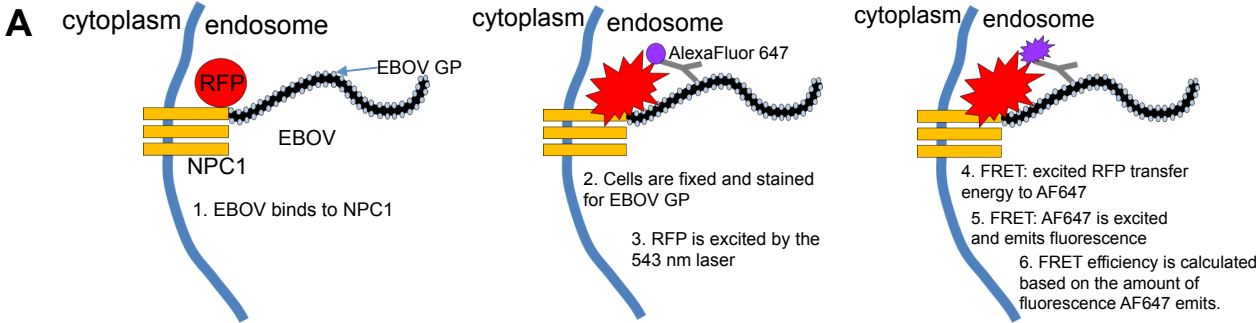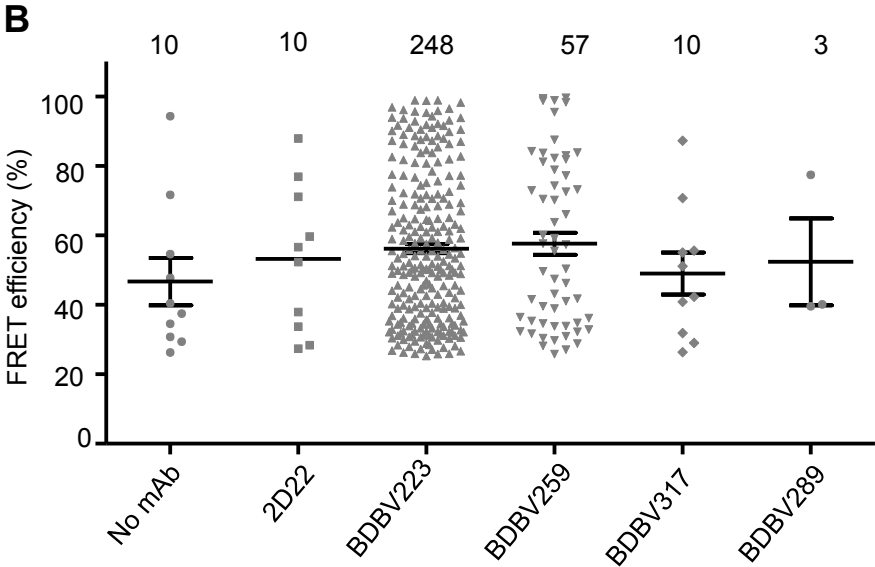

Supplement: S8 Fig — A. Schematic representation of FRET for analysis of the binding of GP to NPC1 in the late endosomes. B. FRET efficiency, which represents a percentage of the maximal amount of fluorescence emitted by acceptor fluorophore when excited by the donor fluorophore in the presence of the indicated mAb. Cells transfected with NPC1-RFP were inoculated with EBOV/BDBV-GP_no eGFP in the presence or absence of mAbs, fixed and stained for GP. Each symbol represents an individual FRET positive event. Horizontal lines correspond to the average values of FRET positive events ± SE. The numbers of FRET positive events are shown on the top of each group. Comparison of FRET efficiency to no mAb control showed no statistical significance (Factorial ANOVA, Fisher LSD test). (PDF) [file ppat.1007204.s008.pdf]

**S9 Figure**

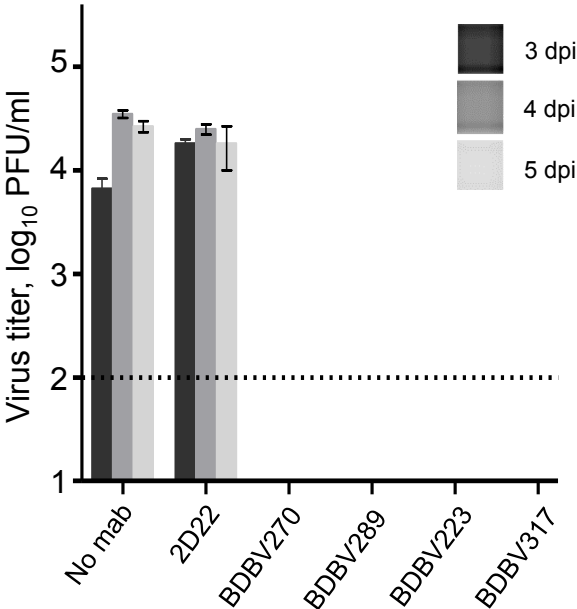

Supplement: S9 Fig — Inhibition of cell-to-cell virus transmission by mAbs: titration of virus in supernatants. Supernatant aliquots were harvested from co-cultures of THP-1 and Vero-E6 cells on days 3–5 after the infection of monocytes and titrated on Vero-E6 cell monolayers. Mean values of triplicate samples ± SE are shown. The limit of detection (2 log10) is indicated by the dotted line. (PDF) [file ppat.1007204.s009.pdf]

## S10 Figure

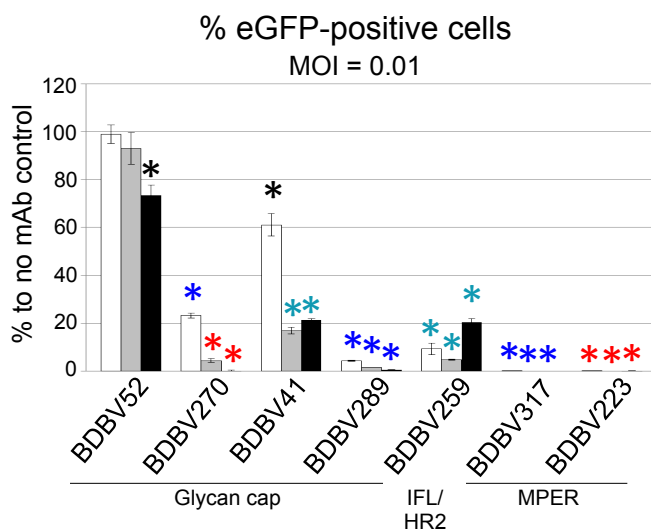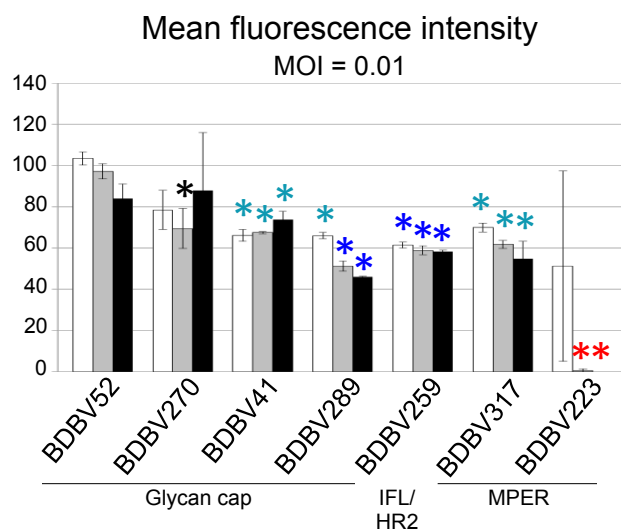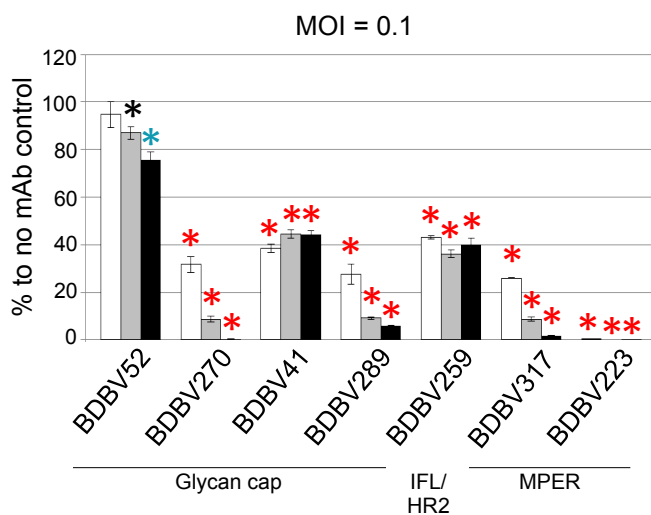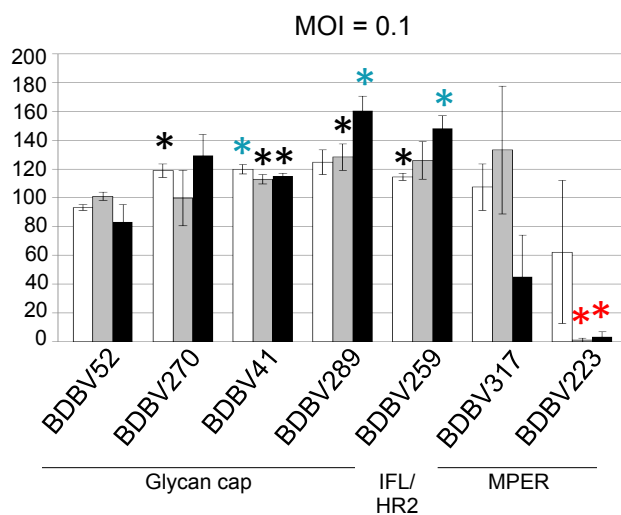

□ 1 µg/ml  
 ■ 10 µg/ml  
 ■ 100 µg/ml

\*  $p < 0.05$   
 \*  $p < 0.01$   
 \*  $p < 0.001$   
 \*  $p < 0.0001$

Supplement: S10 Fig — Vero-E6 cells with various mAb concentrations in medium were inoculated with EBOV/BDBV-GP at MOI of 0.01 PFU/cell (top) or 0.1 PFU/cell (bottom), incubated for 48 hours, fixed and analyzed by flow cytometry. Bars show percentage of reduction of the numbers of eGFP+ cells (left) or MFI (right) compared to no mAb control, mean values of triplicate samples ± SE. P values were calculated by unpaired Student’s t-test, compared to no mAb control. (PDF) [file ppat.1007204.s010.pdf]

S11 Figure

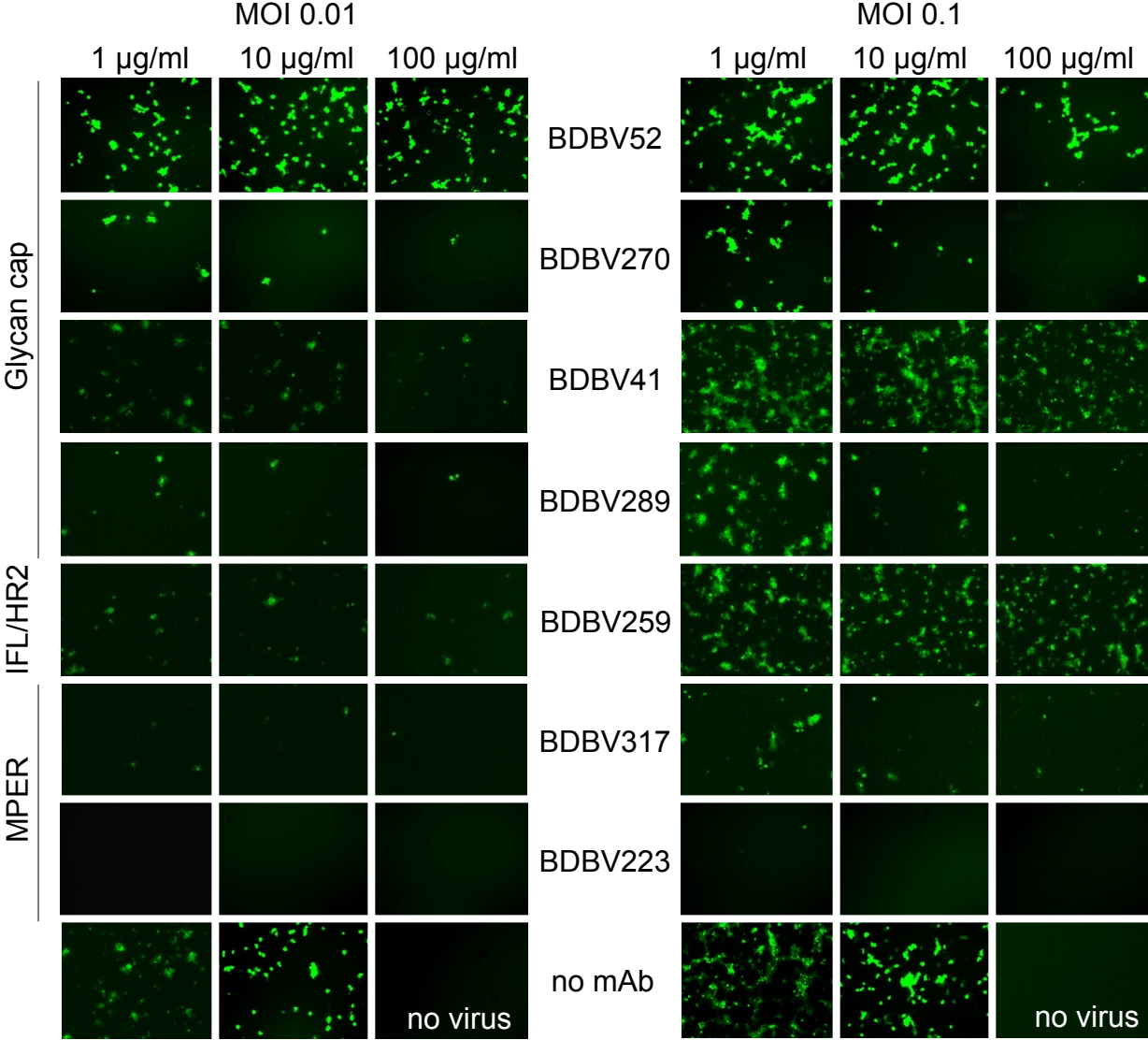

Supplement: S11 Fig — Vero-E6 cells with various mAb concentrations in the medium were inoculated with EBOV/BDBV-GP at MOI of 0.01 PFU/cell (left) or 0.1 PFU/cell (right), incubated for 48 hours and analyzed by UV microscopy. (PDF) [file ppat.1007204.s011.pdf]

S12 Figure

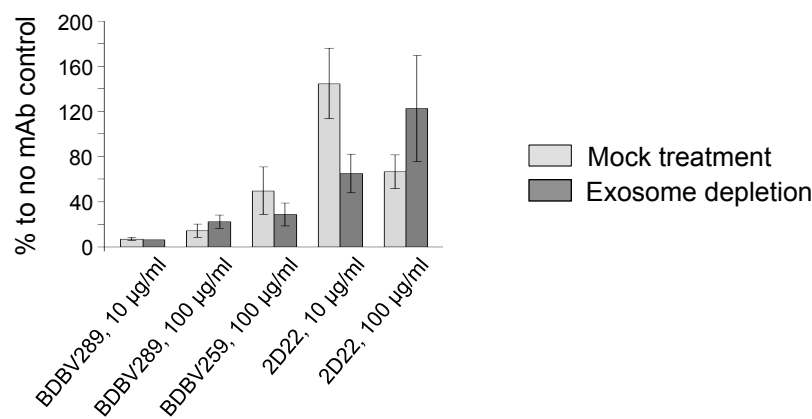

Supplement: S12 Fig — Exosome depletion does not affect the content of viral RNA in cell supernatants. Bars indicate viral RNA load, determined by digital droplet RT-PCR, in supernatants of cells infected with EBOV/BDBV-GP with or without exosome depletion. Mean values normalized to no-mAb control based on triplicate samples ± SE. (PDF) [file ppat.1007204.s012.pdf]

S13 Figure

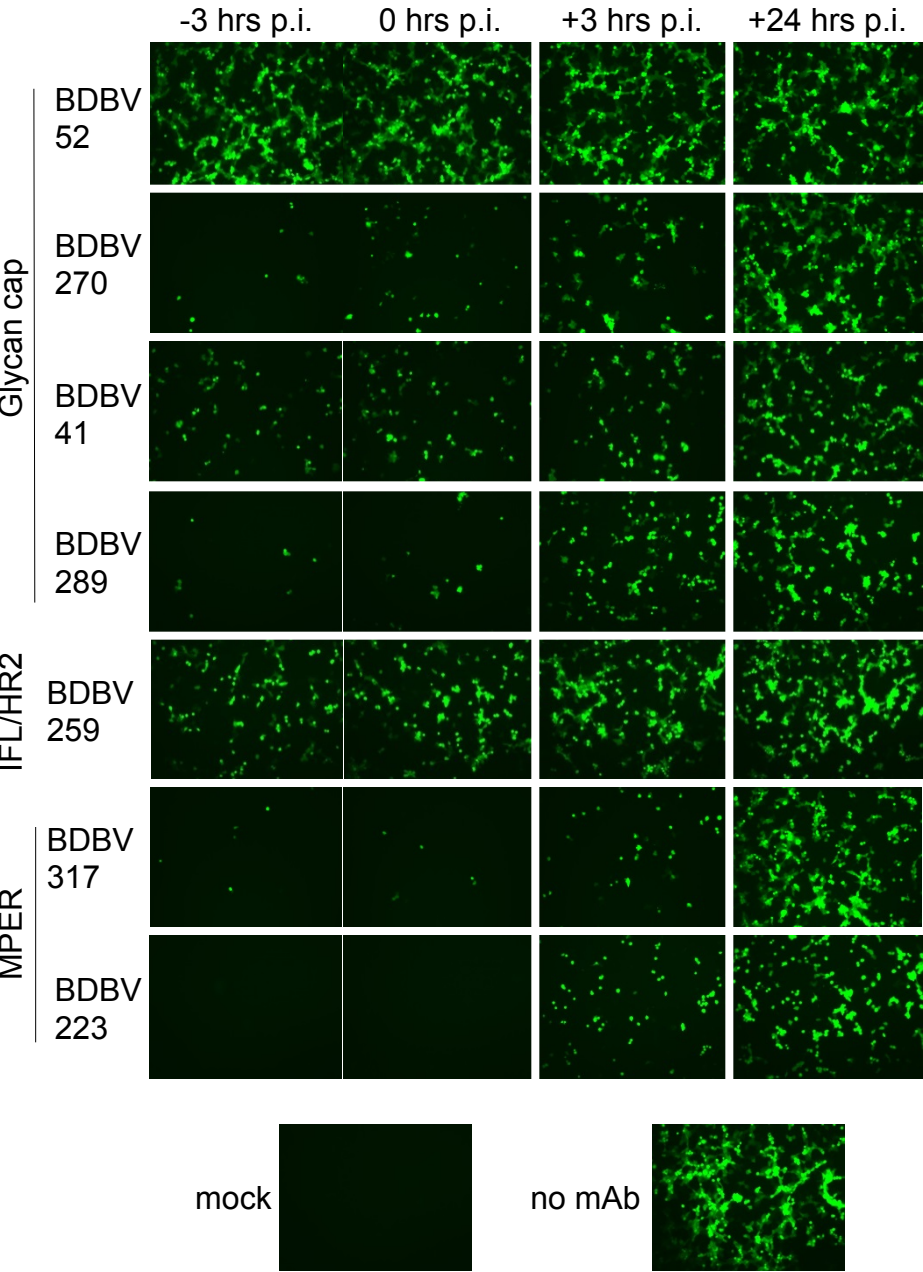

Supplement: S13 Fig — Vero-E6 cells were inoculated with EBOV/BDBV-GP at MOI of 0.1 PFU/cell, and mAbs were added at the indicated time points with final concentration of 100 μg/ml. UV microscopy photographs of cell culture monolayers taken at 48 hours after infection. (PDF) [file ppat.1007204.s013.pdf]

S14 Figure

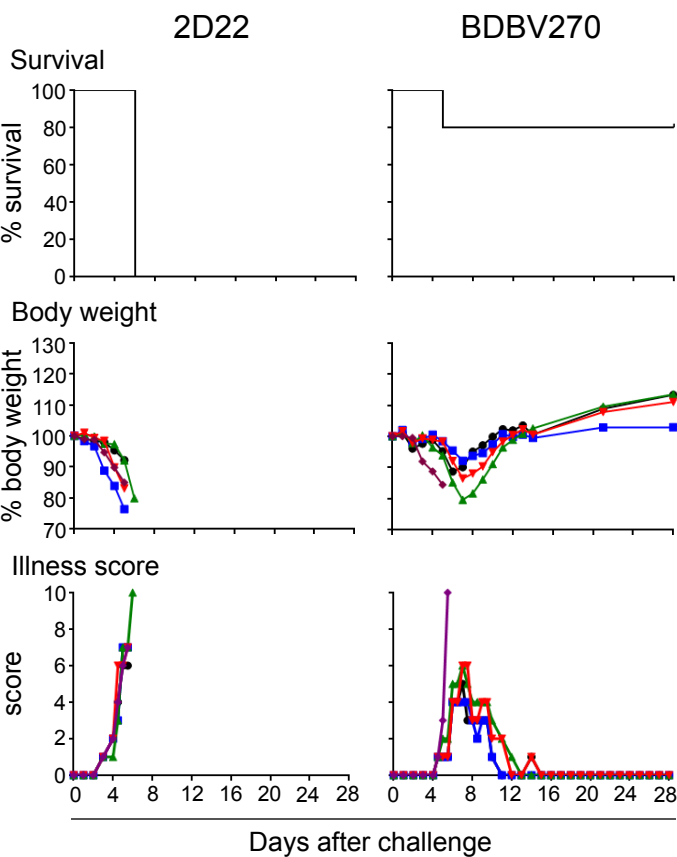

Supplement: S14 Fig — Groups of mice at five animals per group were injected with 100 μg of an irrelevant mAb 2D22 or BDBV270 by the intraperitoneal route at 24 hours after EBOV challenge. Kaplan-Meier survival curves (p = 0.0644, Mantel-Cox test), body weight and illness score curves are shown. (PDF) [file ppat.1007204.s014.pdf]

## S15 Figure

**A**

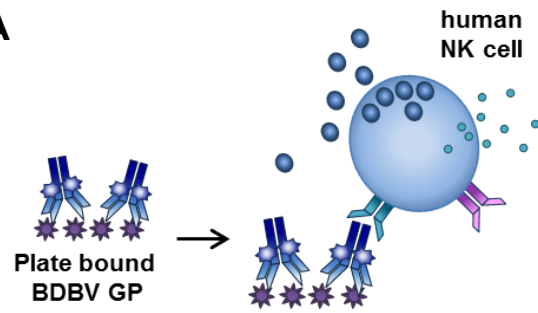

**B**

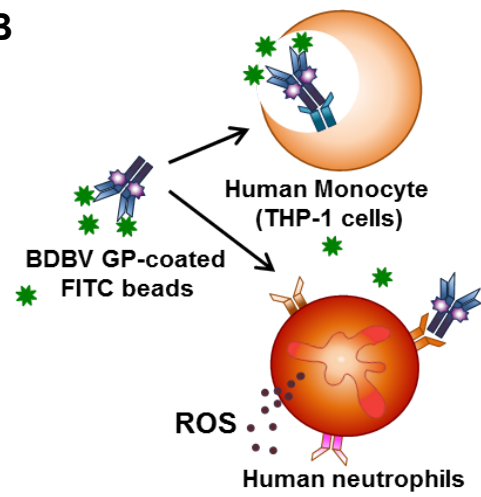

Supplement: S15 Fig — Schemes of NK cell activation (A, related to Fig 5A, 5B and 5C) and phagocytosis assays (B, related to Fig 5D and 5E). (PDF) [file ppat.1007204.s015.pdf]

## S16 Figure

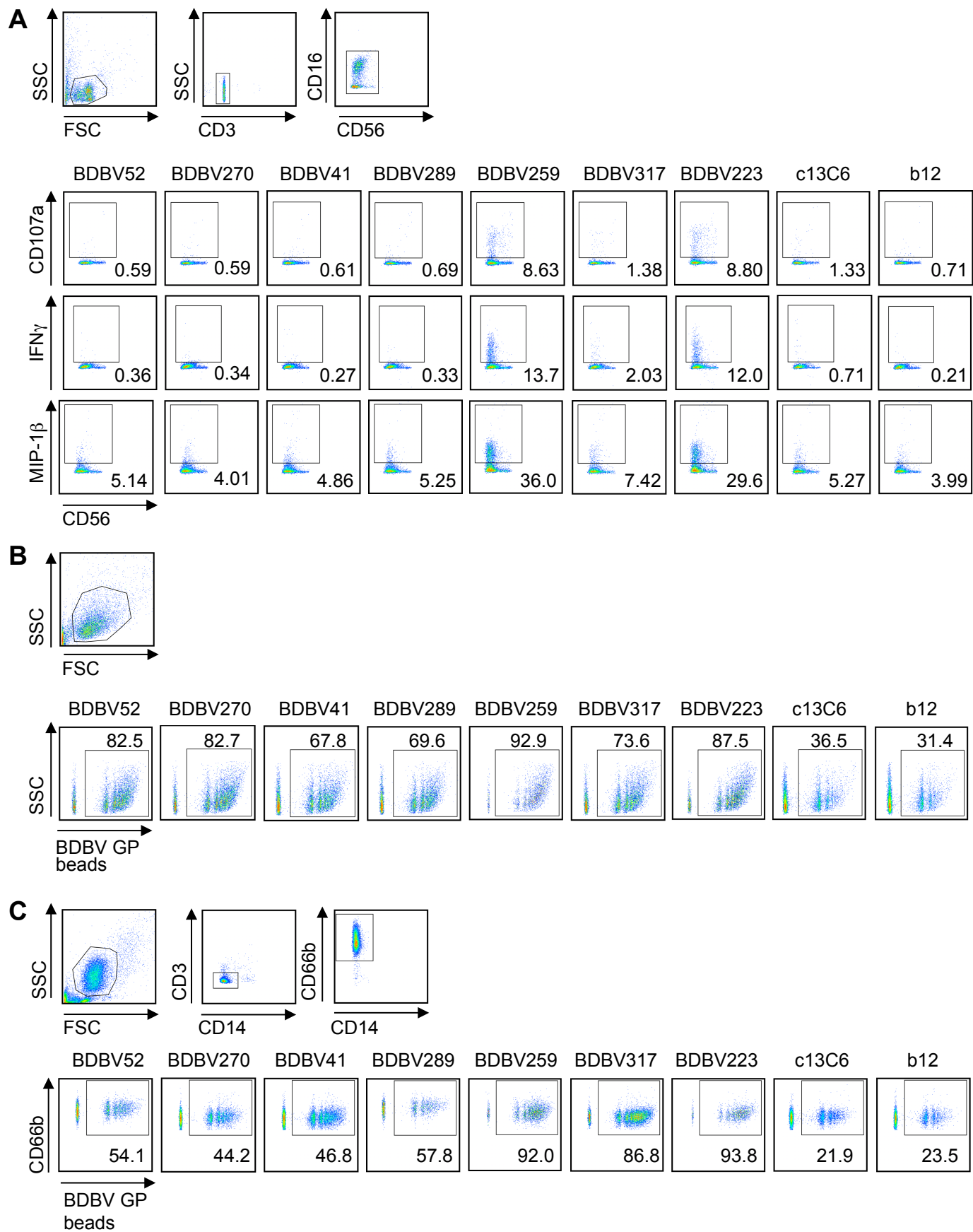

Supplement: S16 Fig — Gating strategy for analysis of antibody dependent activation of NK cells (A, related to Fig 5A, 5B and 5C), antibody dependent monocyte phagocytosis (B, related to Fig 5D) and antibody dependent neutrophil phagocytosis (C, related to Fig 5E). Representative flow cytometry plots of the indicated monoclonal antibodies in response to recombinant BDBV GP. (PDF) [file ppat.1007204.s016.pdf]
